# Direct and Observed Joint Attention Modulate 9-Month-Old Infants’ Object Encoding

**DOI:** 10.1162/opmi_a_00114

**Published:** 2023-11-27

**Authors:** Maleen Thiele, Steven Kalinke, Christine Michel, Daniel B. M. Haun

**Affiliations:** Department of Comparative Cultural Psychology, Max Planck Institute for Evolutionary Anthropology, Leipzig, Germany; Department of Early Child Development and Culture, Leipzig University, Leipzig, Germany; SRH University of Applied Health Sciences, Gera, Germany

**Keywords:** infancy, observational social learning, joint attention, object encoding, memory, eye-tracking

## Abstract

Sharing joint visual attention to an object with another person biases infants to encode qualitatively different object properties compared to a parallel attention situation lacking interpersonal sharedness. This study investigated whether merely observing joint attention amongst others shows the same effect. In Experiment 1 (first-party replication experiment), *N* = 36 9-month-old German infants were presented with a violation-of-expectation task during which they saw an adult looking either in the direction of the infant (eye contact) or to the side (no eye contact) before and after looking at an object. Following an occlusion phase, infants saw one of three different outcomes: the same object reappeared at the same screen position (no change), the same object reappeared at a novel position (location change), or a novel object appeared at the same position (identity change). We found that infants looked longer at identity change outcomes (vs. no changes) in the “eye contact” condition compared to the “no eye contact” condition. In contrast, infants’ response to location changes was not influenced by the presence of eye contact. In Experiment 2, we found the same result pattern in a matched third-party design, in which another sample of *N* = 36 9-month-old German infants saw two adults establishing eye contact (or no eye contact) before alternating their gaze between an object and their partner without ever looking at the infant. These findings indicate that infants learn similarly from interacting with others and observing others interact, suggesting that infant cultural learning extends beyond infant-directed interactions.

## INTRODUCTION

During the first year after birth, infants develop foundational abilities enabling them to learn culturally shared knowledge from others. Most studies on social learning in infancy have focused on infant-directed social interactions and less on infants’ observational learning from others without being addressed directly. However, a growing body of research suggests that third-party observation represents a central childhood learning context, and that ignoring this learning context risks underestimating the diversity and flexibility of infants’ social learning. First, findings from cross-cultural and anthropological studies show systematic variation in the extent to which infants encounter child-directed interactions, and in how much they are expected to learn from observing others (Correa-Chávez & Rogoff, 2009; Gaskins & Paradise, 2010; Keller, 2007; Paradise & Rogoff, 2009; Shneidman, Gaskins, & Woodward, 2016; for a review see Shneidman & Woodward, 2016). Second, in cultural contexts where children typically experience high levels of direct pedagogy, toddlers around their second birthday imitate actions and learn novel word labels equally well when directly addressed as when they observe a social interaction between third parties (Akhtar et al., 2001; Floor & Akhtar, 2006; Gampe et al., 2012; Matheson et al., 2013; Nielsen et al., 2012; Shneidman et al., 2014). To gain a comprehensive picture of the multiple facets of social learning in the first year of life, studies on observation-based forms of learning are needed in addition to participatory forms. Here, we investigated whether joint attention, a social context instrumental for cultural learning, modulates 9-month-old German infants’ object encoding similarly across self-experienced (“first-party”) and observed (“third-party”) social interactive settings.

### First-Party Joint Attention Supports Early Cultural Learning from Infancy Onwards

Many theoretical accounts on the ontogeny of cultural learning have stressed joint attention—the representation of the shared and mutually recognized attention between two people and an object—as a hallmark of human social cognition and an important context for the transmission of culture-specific knowledge (Barresi & Moore, 1996; Bruner, 1995; Moore, 2007; Tomasello, 1995; Tomasello et al., 2005). More than attending to an object at the same time (“parallel attention”), “truly” *joint* attention has been theorized to involve intersubjective sharedness—a shared psychological space enabling referential communication and knowledge transmission (Tomasello & Carpenter, 2007). Infants begin to engage in joint attention during the second half of the first year of life (Carpenter et al., 1998; Striano & Bertin, 2005), potentially universally across cultural contexts (Callaghan et al., 2011). The most commonly used behavioral marker of infants’ emerging awareness of interpersonal sharedness within triadic interactions is the presence of eye contact before (“initiation look”) and after (“sharing look”) looking at an object together (e.g., Bakeman & Adamson, 1984; Carpenter & Liebal, 2012). More recent research has shown that interpersonal sharedness can also be established through physical modalities, including interpersonal proximity or touch (Abels, 2020; Botero, 2016; Little et al., 2016; Yu & Smith, 2013).

Empirical evidence for the supportive effect of joint attention for cultural learning comes mainly from studies with urban, middle-class populations from the USA, the United Kingdom, Europe, and Australia (the “Global North”). They reveal that adult-child interactions involving eye contact and mutually shared attention to an object (vs. parallel attention to an object lacking eye contact) support children’s learning of object-related actions, word labels, or tool-use functions during the second year of life (Hirotani et al., 2009; Matheson et al., 2013; Sage & Baldwin, 2011). Moreover, studies focusing on the early stages of memory formation suggest that the learning-facilitating effect of joint attention develops well before children engage in action imitation or word learning. Starting at around 4 months of age, infants show increased object encoding when seeing an adult looking at an object after looking in the direction of the infant (Hoehl et al., 2014; Reid & Striano, 2005; Wahl et al., 2013). Slightly later, infants become increasingly sensitive to the presence of eye contact when encoding a novel object in the context of triadic social interactions: 7- and 9-month-old infants, but not 4- and 5-month-olds show increased object encoding when looking at an object jointly with an adult before and after engaging in eye contact compared to when looking at it in parallel, without eye contact (live-interactive setting: Cleveland et al., 2007; Cleveland & Striano, 2007; screen-based setting: Okumura et al., 2020; Thiele et al., 2021b, Experiment 1).

Important for the focus of this study, some evidence suggests that the early emerging memory-enhancing effect of joint attention is highly specific in that it modulates not only how much, but indeed *how* infants represent and encode a mutually attended object. Instead of generally increasing the processing of all available visual information, first-party joint attention biases 9-month-old infants’ memory selectively toward those object properties allowing an infant to recognize an object outside a learning situation in the future or to detect other objects of the same kind. In the first study revealing this memory effect in preverbal infants, Yoon et al. (2008) introduced it as the “communication-induced memory bias”. Based on assumptions of the Natural Pedagogy account (Csibra & Gergely, 2006, 2011; Csibra & Shamsudheen, 2015), the authors theorized that the presence of a communicative context enhances infants’ processing of *internal* object features, such as surface features specifying identity or kind, over transient *external* object features, such as spatiotemporal features like location or size. To test this hypothesis, 9-month-old UK infants were presented with a novel object embedded within a communicative or a non-communicative social scene, using a screen-based violation of expectation (VoE) task (Yoon et al., 2008). In the videos depicting a communicative scene, a female actor pointed and gazed at a visible object after addressing the infant via eye contact, waving gestures, and infant-directed speech. In the non-communicative videos, the actor reached for an object while gazing at it without addressing the infant. Following each video, the scene was occluded in a way that the empty space between the two objects remained visible to the infant (i.e., a location change outcome was principally impossible). Then, infants were presented with one out of three possible outcomes: the object had (a) changed its’ location on screen (location change), (b) was replaced by an entirely novel object (identity change), or (c) remained unchanged (no change). In line with their theoretical assumptions, Yoon et al. (2008) found a selective memory bias for identity-relevant information in the communicative condition: Infants looked longer at identity changes than at no change or location change outcomes (“identity bias”). In contrast, in the non-communicative condition, infants looked longer at location changes than at identity change or no change outcomes (“location bias”).

A study by Okumura et al. (2016) partially replicated the findings by Yoon et al. (2008) with 9-month-old Japanese infants. In deviation from the original study, Okumura et al. (2016) focused on gaze cues only to disentangle the influence of joint *visual* attention from the multitude of social cues presented in the stimuli by Yoon et al. (2008). Moreover, infants were tested in a live-interactive study procedure instead of a screen-based setting. Depending on the condition, an adult experimenter either engaged the infant in eye contact (joint attention) or looked at the ceiling (non-joint attention) before and after looking at a visible object. Then, the scene was occluded before one of the same three outcomes was revealed as in the study by Yoon et al. (2008) (location change, identity change, no change). In deviation from the original study, however, Okumura et al. (2016) covered the entire scene during the delay phase (i.e., a subsequent object change outcome was in principle possible). In line with the communicative condition by Yoon et al. (2008), Okumura et al. (2016) found that infants in the joint attention condition looked longer at identity changes compared to no change and location change outcomes, suggesting that gaze cues alone can bias infants’ memory toward encoding and retaining recognition-relevant object features. In the non-joint attention condition, in contrast, infants encoded identity *and* location changes.

A third study by Silverstein et al. (2019) failed to replicate the findings of Yoon et al. (2008). The authors used conceptually similar stimuli as in the original study and the same manual coding procedures to retrieve the dependent measures. Still, they could not replicate the communication-induced memory bias in two experiments. Instead, 9-month-old UK infants either looked longer at identity changes regardless of condition (Experiment 1) or showed no memory biases at all (Experiment 2; for a detailed discussion of the failed replication see Silverstein et al., 2019).

In summary, previous research suggests that first-party joint attention provides a supportive context for cultural learning from infancy onwards. By the age of 9 months, infants represent, perceive, and memorize a novel object in a particular and potentially qualitatively different way when it is embedded within a joint attention interaction than when encountering the same object in a social situation lacking interpersonal sharedness.

### Third-Party Joint Attention as a Learning-Enhancing Social Context

In the action imitation and word learning literature, one observational learning context that is receiving increasing attention is the observation of others’ social interactions (“third-party interactions”). Most studies investigating social learning in third-party interaction contexts have compared children’s learning from first-party pedagogical interactions with their observational learning from third-party pedagogical interactions (Akhtar, 2005; Correa-Chávez & Rogoff, 2009; Gampe et al., 2012; Matheson et al., 2013). Little attention has been devoted to what factors *within* an observed interaction situation contribute to children’s observational learning. Some early evidence suggests that, like in first-party interactions, third-party joint attention may be supportive in guiding children’s learning about novel objects. At around 18 months of age, German and Japanese toddlers are sensitive to the presence or absence of intersubjective sharedness when observing two people attending to an object (Gräfenhain et al., 2009; Meng et al., 2017), Swedish toddlers rely on social engagement to infer joint action goals between third parties (Fawcett & Gredebäck, 2015), and US American toddlers use cues of intersubjective sharedness to acquire conventional knowledge: 18-month-olds learn a novel object label through eavesdropping a conversation, but only when the overheard interlocutors attend jointly to a referenced object during labeling, not when one of the partners is distracted with another activity (Fitch et al., 2020).

Already during the second half of the first year of life, infants in the Global North develop foundational abilities and preferences enabling them to detect, process, and sustainedly observe dyadic social interactions between third parties (Farris et al., 2022; Galazka et al., 2014; Goupil et al., 2022; Handl et al., 2013; Thiele et al., 2021a). During the same period, infants develop an increasing understanding of third-party interactions, including the turn-taking dynamic between two social partners (Augusti et al., 2010; Bakker et al., 2011; Beier & Spelke, 2012), their communicative intentions (Thorgrimsson et al., 2014, 2015), their reciprocal exchange relations (Tatone et al., 2021), and their (shared) action goals (Elsner et al., 2014; Gredebäck & Melinder, 2010). In addition, some evidence indicates that observing others’ interactions actuates an internal attentional stance in infants, potentially facilitating the encoding of novel information. Compared with an observed back-to-back scene, videos depicting a third-party face-to-face interaction elicit increased pupil dilation in 12-month-old French infants (Gustafsson et al., 2016), facilitate extracting statistical regularities of gesture sequences and emotional facial expressions in 12-month-old Italian infants (Mermier et al., 2022; Quadrelli et al., 2020), and enhance associative visual learning in 13-month-old Swedish infants (Thiele, Hepach, Michel, Gredebäck et al., 2021).

Together, these findings suggest that infants in the first year of life develop abilities beneficial for observational learning in the context of third-party interactions. However, what remains largely unknown is whether infants already *learn* from such observations about novel objects and, more specifically, whether observations of third-party joint attention support their referential learning. To our knowledge, only one previous study has addressed this question, focusing on object encoding as an early stage of object-related learning (Thiele et al., 2021b). Based on a two-by-two design, 9-month-old German infants saw four kinds of videos showing two adults engaging in eye contact (or looking away from one another) before shifting their gaze toward (or away from) a visible object. As an indicator of object encoding, Thiele et al. (2021b) measured the infants’ novelty preference in a subsequent preferential-looking test during which the previously familiarized object reappeared next to an unfamiliar novel object. Relatively longer looking times at the novel object were interpreted as an increased previous encoding of the familiarized object (see also Reid & Striano, 2005). Infants showed a novelty preference only after observing two adults looking at an object following mutual eye contact (joint attention), but not after any other condition. In line with previous first-party studies (e.g., Cleveland & Striano, 2007; Okumura et al., 2020), the authors found the same result pattern in a matched first-party experiment, in which 9-month-old infants were directly addressed by an adult on screen.

The study by Thiele et al. (2021b) shows that even in a socialization context where child-directed face-to-face interactions represent the predominant social learning setting, infants begin to learn from others based on observing their social interactions. Moreover, the study demonstrates that first- *and* third-party joint attention enhance infants’ encoding of a referenced object, supporting the idea that observing joint attention may have a similar effect on learning as participating in joint attention. However, what remains unclear is what *kinds* of information infants represent and process about an object when seeing two people attending jointly to it, and whether this differs from an observed social learning situation lacking interpersonal sharedness. Inspired by previous first-party studies revealing a selective memory bias toward encoding and memorizing recognition-relevant object features during infant-directed referential communication (Yoon et al., 2008; but see Silverstein et al., 2019) and joint visual attention (Okumura et al., 2016), affirmative evidence would underline the relevance and flexibility of a shared psychological space for cultural learning in infancy.

### The Current Study

This study aimed to conceptually replicate the selective memory bias for identity information during first-party joint visual attention and investigate whether it extends to an observational setting where infants encounter a joint visual attention situation from a third-party perspective. We conducted two experiments to directly compare infants’ responses in a first-party context (Experiment 1, conceptual replication of Okumura et al., 2016) with a third-party context (Experiment 2). In Experiment 1, 9-month-old German infants were presented with a VoE task during which they saw two kinds of videos showing an adult gazing at an object. We systematically manipulated whether the object-related gaze shift was embedded within a joint attention context. For this purpose, the adult looked either in the direction of the infant (eye contact) or to the side (no eye contact) before and after looking at an object. After each video, the scene was occluded before it revealed one of three different outcomes: the same object the infant had just seen reappeared at the same screen position (no change, baseline), the same object reappeared at a novel screen position (location change), or a completely novel object was shown at the same screen position (identity change). As the dependent measure, we compared infants’ looking times at outcomes containing an unexpected change (location or identity change) with the outcome containing no change. We made the following general assumptions according to the logic that infants tend to look longer at events that violate their expectations: If infants look longer at the change outcomes compared with the no-change baseline, we assumed that they had retained information about that feature in their memory. If infants find one type of unexpected change more interesting or surprising than another (location or identity), we assumed that they would respond with increased attention and longer looking time to this outcome (comparison between outcomes).

As preregistered, we hypothesized that the following result patterns would be indicative of a communication-induced identity bias. Our predictions were based on two previous studies revealing such a memory bias in preverbal infants (Okumura et al., 2016; Yoon et al., 2008). In a joint attention scene involving eye contact and referential gaze to an object, infants should show an identity bias, that is, increased looking times at identity changes compared to the baseline and compared to location changes (in line with Okumura et al., 2016; Yoon et al., 2008). In non-coordinated parallel attention scenes, two patterns would indicate a communication-induced memory bias: Infants should either (a) show a location bias, that is, increased looking times at location changes over identity changes and compared to the baseline (Yoon et al., 2008) or they should (b) show both a location and identity bias, that is, increased looking times at location changes and identity changes compared to the baseline, with no difference between the change outcome conditions (Okumura et al., 2016). The reason for preregistering two possible result patterns in the parallel attention conditions was to account for two possible underlying mechanisms previously proposed to drive the memory bias in infants: a resource-saving mechanism according to which infants selectively invest their limited resources into the most relevant object properties within a given social situation (result pattern (a), Yoon et al., 2008) or a disruption effect of spatial-temporal information over identity-relevant information in communicative contexts (result pattern (b), Okumura et al., 2016).

In Experiment 2, 9-month-old infants were presented with the same task, but the videos showed two adults establishing eye contact (or no eye contact) before alternating their gaze between an object and one another. Our hypotheses were the same as in Experiment 1. Each infant participated in only one of the two experiments. We did not preregister any predictions regarding the comparison between first- and third-party context.

## EXPERIMENT 1: FIRST-PARTY CONTEXT

### Methods

Ethical approval for the design and procedure of this study was provided by the Ethics Council of the Max Planck Society. We preregistered our hypotheses, methods, procedures, and the data analysis plan for this experiment prior to data collection on the Open Science Framework (OSF; link: https://osf.io/t4yqj/). Video examples, eye-tracking raw data, and R scripts for pre-processing and analyzing the data are available at the same link on the OSF. We used the study by Okumura et al. (2016) as the primary reference for decisions such as the timing of the stimuli, the overall procedure of the task, and inclusion criteria, because this study focused on gaze cues only and was therefore most comparable with our study.

#### Participants.

As preregistered, 36 typically developing infants from Leipzig (Germany), between 9 months, 0 days and 10 months, 0 days of age were included in the final sample of Experiment 1 (*n* = 16 female; *M* = 286.16 days, *SD* = 8.63 days). Data from five additional children were excluded because they were younger than the age inclusion criterion (*n* = 1), born preterm (*n* = 1), or because they did not provide the minimum amount of one valid trial per condition (*n* = 3). The sample size was planned based on a simulation-based a priori power analysis using the raw data by Okumura et al. (2016) to estimate the effect sizes of the simulated data (for details see section S1 in the Supplementary Materials). All included infants were born full-term (> 37 weeks). They were recruited on a voluntary basis via phone from the database of the Max Planck Institute for Evolutionary Anthropology. Children in this database come from Leipzig or surrounding areas, an urban Central-European, industrialized context. We did not collect individual data regarding the participants’ socioeconomic background, but families in this database typically come from mixed, mainly mid to high socioeconomic backgrounds. In Germany, infants in the tested age range typically spend their days at home in the presence of their primary caregiver. The prototypical socialization context is a child-centered setting during which infants experience high levels of direct pedagogy involving face-to-face interactions and object play. We obtained written informed consent from one caregiver of each infant before the testing session started. All participants received a small gift as thank you for their participation.

#### Design and Procedure.

The testing took place at the Max Planck Institute for Evolutionary Anthropology in Leipzig (Germany). All infants participated in a VoE task during which they sat in front of a screen on their parent’s lap. The task was presented on a 24.1″ monitor with 94 ppi and 1920 × 1080 screen resolution. To run the experiment and to record infants’ gaze movements, a Tobii eye-tracker (TX120, Tobii Technology, Stockholm, Sweden) and Tobii eye-tracking software (Tobii Studio version 3.4.8.1348) was used. Data was recorded separately for the left and the right eye at a sampling frequency of 60 Hz. We used a five-point calibration procedure to calibrate the eye-tracker to the participant’s eyes. The total duration of the Experiment was approximately 10 minutes, the whole visit at the lab around 30 minutes.

First, infants saw two pretest trials (as in Okumura et al., 2016; Silverstein et al., 2019; Yoon et al., 2008). These trials aimed to familiarize the infants with the actors, the action-occlusion-outcome structure of the test trials, and the two object positions. The pretest trials contained the same three phases as the test trials: an action phase showing a female actor gazing at a novel object (15 s), a delay phase during which the scene was occluded (3 s), and an outcome phase during which a single object was shown (15 s or until the infant looks away for two consecutive seconds). Before the action and outcome phase started, a blinking star was presented in the center of the screen to capture the infant’s attention and centralize their gaze (stimulus examples pretest trials: https://osf.io/v2cfx). Moreover, it decreased the likelihood of trial exclusion due to missing data in the outcome phase.

Following the pretest trials, each infant was presented with 12 test trials. Based on a 2 × 3 design, we manipulated the eye contact between the actor and the infant in the action phase (eye contact or no eye contact) and the object change in the outcome phase (no change, identity change, location change). Each infant saw two trials per each of the resulting six conditions. This deviated from previous studies where only one trial was presented in each condition. We decided to present two trials per condition to maximize the number of available data points, increase statistical power, and decrease dropouts due to our trial inclusion criterion. The trials were presented in four blocks with three trials each. Within a given block, each trial presented a different outcome. In the first test trial block, we counterbalanced the order of outcome and eye contact conditions across infants in a way that an equal number of infants saw an identity change, a location change, and a no change first, and an equal number of infants saw a trial of the “eye contact” and the “no eye contact” condition first. In the remaining three trial blocks, the order of outcome and eye contact conditions was pseudo-randomized. No outcome or eye contact condition occurred more than twice in a row. After every block, infants were presented with a 4-second kaleidoscope video with a soothing melody to maintain their attention.

#### Stimuli.

We created the videos in a way that the timing and content were as similar as possible to the live-interactive study procedure by Okumura et al. (2016) while keeping the visual details consistent with the third-party videos in Experiment 2 (for details see section S2 in the Supplementary Materials). For counterbalancing, we created six experimental orders in the eye-tracking software (link to counterbalancing sheet: https://osf.io/xqem3). Every participant was randomly assigned to one of these orders. Each order was presented to six infants.

##### Action Phase.

**Figure 1.**
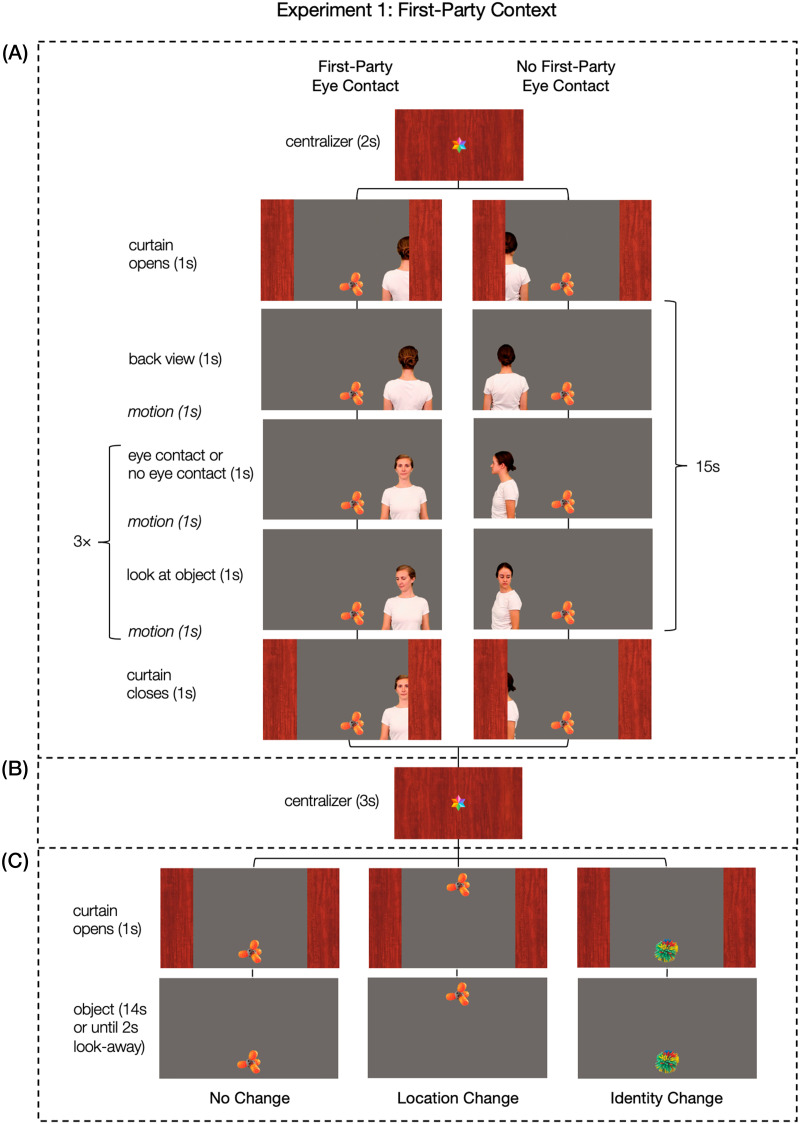
**Exemplary Sequence and Timing of one Test Trial in Experiment 1.** *Note*. Every trial consisted of (A) an action phase (15 s), (B) a delay phase (3 s), and (C) an outcome phase (15 s or until the infant looked away). Before the action and the outcome phase, an attention-getting animation (blinking star) was presented in the center of the screen. The position of the actor (left or right) and the position of the object in the action phase (top or bottom) was counterbalanced. Figure S3 in the Supplementary Materials illustrates the counterbalancing version in which the object was positioned at the top screen position in the action phase.

The action phase began with a schematic curtain opening during one second. The movement of the curtain was accompanied by attention-getting sounds. In the test trials, a novel object was introduced either in a joint attention context including eye contact, or a non-joint attention context without eye contact. To manipulate the eye contact between actor and infant, we used the movement of the actor’s body (turning toward the child or to the side), the relative positioning of their body (facing the child or averted), and their gaze direction (looking toward the child or to the side). All action phase videos had a total duration of 15 seconds after the curtain was completely open. The timing was identical over all conditions and videos (see Figure 1): In the “eye contact” conditions, an adult woman was shown in back view (1 s) before she turned toward the infant (1 s) and remained in this position (1 s). Then, she shifted her gaze toward a visible object displayed at the top or bottom of the screen (1 s), looked at it (1 s), shifted her gaze back in the initial position (1 s), and remained in this position (1 s). The actor repeated the turn-taking gaze pattern between the infant and the object for two more times ending in a look toward the infant. She kept a neutral facial expression and remained silent during the entire sequence (stimulus examples “eye contact” condition: https://osf.io/84psu). The timing in the “no eye contact” conditions was the same as in the “eye contact” conditions, but the woman never gazed in the direction of the infant. Instead, like in the “no eye-contact - look at object” condition in Thiele et al. (2021b), she turned to the side before shifting her gaze to the object, and then alternating her gaze between the object and the initial position (stimulus examples “no eye contact” condition: https://osf.io/fmkvh).

Each infant saw two female actors in the videos of the action phase. One actor performed in all six trials of the “eye contact” condition and the other actor performed in all six trials of the “no eye contact” condition. Across participants, each of the two actors engaged equally often in eye contact and no eye contact. The actor’s position in the action phase (left or right from the object) was counterbalanced within eye contact conditions, outcome condition, and child. Each infant saw the actor three times on the left side and three times on the right side within each eye contact and outcome condition. The actor’s position in the first two test trials was counterbalanced across children such that an equal number of children saw the actor on the right side and on the left side on the first test trial (and on the correspondingly other side on the second test trial). All possible body and head movements from all actors in all conditions covered an area of 13.8° × 19.0° on both sides of the object, with the head movements covering an area of 10.0° × 10.0°. The objects covered an area of 6.5° × 6.5° (see Figure S1 in the Supplementary Materials for an illustration of the visual arrangement on screen).

##### Delay Phase.

The delay phase began with the curtain closing in front of the action phase video during one second. Then, the scene remained completely occluded for three seconds.

##### Outcome Phase.

The curtain opened during one second, revealing one out of three possible outcomes in the test trials. Infants either saw the same object as in the action phase appearing at the same location on screen (no change, baseline), the same object appearing at a novel location on screen (location change), or a novel object appearing at the same screen location as in the action phase (identity change). The outcome phase concluded when the infant looked off-screen for two consecutive seconds or when 15 s elapsed. To execute the two- second criterion during the testing session, the experimenter manually stopped the time intervals when the infant started turning their gaze away from the screen. When an infant did not look back to the screen for two consecutive seconds, the experimenter started the next trial. To determine the moments of look-aways for our main dependent measures, we used a data-driven approach described in more detail in the Data Processing section below.

The videos in the pretest trials had the identical temporal structure as the videos in the test trials. However, the videos differed in two regards from the test trial videos (as in Okumura et al., 2016; Silverstein et al., 2019; Yoon et al., 2008). First, in the action phase, the actors did not perform any referential looks in the direction of the object. Instead, they turned toward (or away) from the infant before looking *away* from the object (as in the “no look at object” conditions in Thiele et al., 2021b). Second, the post occlusion outcomes did not include any changes in the object’s location or identity. The actors in the pretest trials were the same actors who performed in the videos of the test trials. The actor who looked in the direction of the infant in the pretest phase performed the trials of the “eye contact” condition in the test trials, and the actor who turned to the side in the pretest phase performed the trials in the “no eye contact” condition in the test trials. We counterbalanced the order of eye contact condition and object position during the pretest phase across participants. An equal number of infants saw the “eye contact” condition and the “no eye contact” condition first. The positioning of the actors in the pretest phase was counterbalanced within participants, meaning that each infant saw an actor on both sides. The positioning of the actor in the first test trial was counterbalanced across participants such that an equal number of infants saw the actor on the right and the left side on the first trial.

As objects in the test phase, we used 16 pictures of abstract toys collected for a study by Wahl et al. (2013). Out of the 16 objects, 12 objects were presented as familiar objects in the action phases of the test trials, and 4 additional objects as novel objects in the outcome phases of the “identity change” condition. Across all participants, each of the 16 objects appeared in all six conditions. In the “identity change” condition, each object served as the familiarized object in the action phase and as the novel object in the outcome phase across all participants. This way, each of the 16 objects appeared in both roles across the overall sample.

The position of the object in the action phase (top or bottom) was counterbalanced within infant and conditions in a way that each infant saw an object appearing equally often at the top or bottom screen position in all three outcomes and both social contexts. Across all participants, an equal number of infants saw the object at the top and the bottom screen position in the action phase of the first test trial.

In addition to the objects used in the test phase, we used two objects in the videos of the pretest phase. Across all participants, each of the two objects occurred equally often in the “eye contact” pretest trial and the “no eye contact” pretest trial. The object position in the action phases of the pretest trials was counterbalanced across infants in a way that each object appeared equally often at the top and the bottom screen position. We furthermore counterbalanced across infants at which screen position the object appeared on the first pretest trial. Each infant saw an object appearing at both screen positions during the two pretest trials (top or bottom).

#### Data Processing.

We used R software environment (R version 4.2.3, RStudio version 2023.03.0) for pre-processing and analyzing the data and for setting areas of interest (AOIs). As the main dependent variable related to infants’ VoE response, we measured their looking times in the outcome phase. Only data from the test trials were included. In contrast to previous studies relying on offline manual coding procedures (Okumura et al., 2016; Silverstein et al., 2019; Yoon et al., 2008), we used eye-tracking technology to record the infant’s eye movements and retrieve the dependent measures. Before running the study, we preregistered a data processing approach that aimed at (a) increasing the comparability with previously manually coded data while (b) using the benefits of eye-tracking data, such as the higher spatial resolution and the possibility to extract fixations. To define fixations, we used the Tobii Velocity-Threshold Identification (I-VT) fixation filter with default settings (for details see section S3 in the Supplementary Materials). Data for both the left and the right eye of each participant were averaged. When one eye could not be measured, the data from the other eye was used.

In line with previous studies, we relied on two measures of infants’ VoE response in the outcome phase: their total looking time duration (as in Okumura et al., 2016, preregistered as the main dependent measure in our study) and their first look duration (as in Yoon et al., 2008, preregistered as an exploratory measure in our study). To explore the comparability between our results based on eye-tracking and previous results based on manual coding procedures, we used the openly available eye-tracking raw data by Silverstein et al. (2019). We extracted and analyzed the same dependent measures as in our study and compared the results with Silverstein et al.’s results based on manually coded data. We found that our total looking time duration measure is comparable with the manually coded total looking time response the authors report in their paper (see section S4 in the Supplementary Materials for procedural details and results of this additional analysis).

##### Total Looking Time Duration.

The total looking time duration at the screen was conceptually most similar to the dependent measure used by Okumura et al. (2016). As a measure of the total looking time duration, we used the cumulative length of all fixations within the screen AOI, beginning at the first frame of the curtain opening and ending when the infant looked away for two seconds or after 15 seconds elapsed. To implement the two-second look-away criterion in the eye-tracking raw data, we monitored the time intervals between consecutive screen fixations. If the time interval was longer than 2000 ms, the trial ended and only the data until this time point was included. In addition to infants’ looking time duration at the *screen* (main dependent measure) we repeated our analyses based on infants’ looking time duration at the *object* (preregistered as an exploratory measure). The reason for this additional analysis was to make use of the higher spatial precision possible with automated eye-tracking and explore the influence of AOI size. The results showed the same result pattern as the analysis of looking times at the screen. For the matter of concision, we report the analyses and results for this exploratory measure in section S5 in the Supplementary Materials.

##### First Look Duration.

To increase comparability with the main measure used by Yoon et al. (2008), we calculated the duration of infants’ first looks at the outcome before any looks away. We defined a look at the object as the time interval between the first fixation in the object AOI and the end of the last fixation within the same AOI, including the duration of saccades between fixations (see also “AOI visit duration” described in the Tobii Studio User Manual version 3.4.5). As preregistered, the first look ended when a gaze sample with coordinates outside the object AOI was detected or when the gaze shift latency between two consecutive object fixations was more than 3 *SD*s longer than the median of a child’s individual gaze shift latency within the object AOI across all object-looks and outcome trials. We included the additional gaze shift latency criterion to accommodate for the possibility that the eye-tracker did not detect an outwards moving saccade when an infant moved their gaze away from the screen. In contrast to the manually coded first look measure by Yoon et al. (2008), our eye-tracking measure focused on overt attentional looks at the *object* instead of the entire screen. As we explain in more detail in section S6 of the Supplementary Materials, we decided on this narrower focus to determine the ending timepoint of the first look more accurately in the eye-tracking data.

We extracted all measures for each trial and participant. To accommodate for inaccuracies in calibration, all AOIs were defined 1° visual angle larger than the maximal dimensions of the stimuli. We only included a trial (a) if the infant had looked at least for the duration of one fixation at the object during the outcome phase and (b) if they had looked at the screen during the central parts of the video for at least one fixation (see also Thiele et al., 2021b). As central parts, we counted the looking-to-object (or away-from-object) phase and at least one of the eye contact (or no eye contact) episodes preceding a referential object look, excluding the motion sequences. For the outcome phase inclusion criterion, we decided to focus on object fixations instead of screen fixations to ensure that all included participants would contribute data for all three measures, including the ones relying on gaze data within the object AOI.

Children were only included in the analysis if they provided valid data in at least one trial per condition after being filtered according to these criteria (see also Wahl et al., 2013). On average, infants provided 1.90 valid trials (*SD* = .30) per condition (for detailed valid trial statistics see Table S1 in the Supplementary Materials).

#### Data Analysis.

To investigate whether infants’ looking time response to changes in object identity and location was influenced by the presence of eye contact, we compared the fit of two generalized linear mixed models (GLMMs) with gamma distribution using likelihood ratio tests: a full model and a reduced model. We fitted the models using the R-package *lme4* version 1.1-32 (Bates et al., 2020). We used GLMMs with a non-normal distribution to account for the right-skewed distribution of the looking time data (Lo & Andrews, 2015, for more details see section S1 in the Supplemental Materials). We ran the same model comparison for both dependent measures (total looking time duration at the screen, first look duration at the object). The factor “eye contact” was included as one binary variable (1/0) and the factor “outcome” as two binary dummy variables in both models (identity change: 1/0; location change: 1/0; no change represented by the intercept). The significance of the individual fixed effects was based on likelihood ratio tests comparing the full models with the respective reduced models excluding the individual fixed effects using the *drop1*-function in R with an alpha-level of .05. In section S7 the Supplementary Materials we provide a more detailed description of the models and the model fitting procedure.

##### Full Model.

As main fixed effects, the full model included all possible main effects and interactions between eye contact and outcome. Specifically, we included the interaction between “eye contact and location change”, the interaction between “eye contact and identity change” and the main effects of “location change”, “identity change”, and “eye contact”. As control variables, we included fixed effects for “running trial number” (1–12), “trial number within condition” (1–2), and “object position” (top or bottom). As random effects, we included “subject” as intercept as well as random slopes on “subject” for “running trial”, “trial within condition”, “object position”, and “eye contact”.

##### Reduced Model.

As main fixed effects, we included “eye contact”, “location change”, and “identity change” in the reduced model. We included the same control variables and random effects as in the full model.

We ran the following analyses in addition to the preregistered plan. First, we conducted six pairwise comparisons to compare the looking time response between the three outcomes within the “eye contact” and “no eye contact” condition. All pairwise comparisons were based on the GLMM fitted for the main analysis, using the R-package *emmeans* version 1.8.1-1 (Lenth, 2022). To account for multiple comparisons, the alpha level was adjusted via Bonferroni correction. Second, we explored the influence of infants’ overt attention distribution during the encoding phase on their encoding performance further. We did not find any systematic condition differences that may explain our result pattern in the outcome phase. We present the corresponding analyses and results in the Supplementary Materials in section S5.

### Results

**Figure 2.**
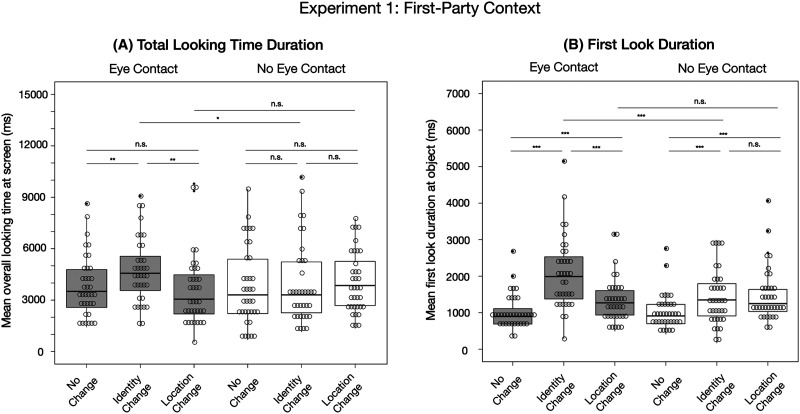
**Results from Experiment 1.** *Note*. Results are based on (A) the total looking time duration at the screen before two consecutive seconds looking away from the screen and (B) the duration of looking time at the object until looking away from the object for the first time. The significances for the pair-wise comparisons within each eye contact condition were retrieved based on the R-package *emmeans*. The significances across eye contact conditions represent the effects of the interactions between “eye contact and location change” and “eye contact and identity change” from the main model comparison.

In the following, we describe the results of our main analyses for both dependent measures. The means and standard deviations are depicted in Table 1 and the significances of the post-hoc pair-wise comparisons are illustrated in Figure 2 (for more statistical details see Tables S2 and S3 in the Supplementary Materials).

**Table 1.** Means and Standard Deviations of the Two Dependent Measures (ms) for the Six Conditions in Experiment 1.

Condition	Duration of total looking time at the screen	Duration of first look at the object
First-party eye contact	3829.22 (*1788.88*)	1020.79 (*476.74*)
No change outcome		
First-party eye contact	4745.54 (*1908.01*)	2078.18 (*978.20*)
Identity change outcome		
First-party eye contact	3598.14 (*2000.38*)	1336.17 (*625.62*)
Location change outcome		
No first-party eye contact	3826.60 (*2303.40*)	1028.00 (*472.07*)
No change outcome		
No first-party eye contact	3913.35 (*2350.92*)	1416.11 (*734.48*)
Identity change outcome		
No first-party eye contact	4048.44 (*1794.07*)	1472.57 (*734.03*)
Location change outcome		

#### Total Looking Time Duration.

The comparison between the full model and the reduced model revealed a *p*-value slightly above the threshold of .05 (*χ*^2^(2) = 5.99, *p* = .05002). We nevertheless explored the significance of the individual fixed effects in the full model to allow for a comparison across all statistical models conducted in this study. We found that the interaction between first-party “eye contact and identity change” had a significant effect on infants’ total looking time duration (*χ*^2^(1) = 4.66, *p* = .03, estimate = 0.26, *SE* = .12), with their looking times at the identity changes being longer in the “eye contact” condition (*M* = 4745.54 ms, *SD* = 1908.01 ms) compared to the “no eye contact” condition (*M* = 3913.35 ms, *SD* = 2350.92 ms). The interaction between first-party “eye contact and location change” did not have a significant effect (*χ*^2^(1) = 0.01, *p* = .91, estimate = 0.02, *SE* = .13). In addition, we did not find significant main effects of “identity change” (*χ*^2^(1) = 0.10, *p* = .75, estimate = 0.03, *SE* = .08), “location change” (*χ*^2^(1) = 0.02, *p* = .88, estimate = 0.01, *SE* = .09), “running trial” (*χ*^2^(1) = 3.13, *p* = .08, estimate = −0.17, *SE* = .09), “trial within condition” (*χ*^2^(1) = 0.98, *p* = .32, estimate = −0.09, *SE* = .09), or “object position” (*χ*^2^(1) = 0.43, *p* = .51, estimate = −0.04, *SE* = .07). The results are illustrated in Figure 2A.

#### First Look Duration.

The comparison between the full model and the reduced model revealed a significant result indicating that at least one of the interactions had an impact on infants’ first look duration at the screen during the outcome phase (*χ*^2^(2) = 21.91, *p* < .001). More specifically, the interaction between first-party “eye contact and identity change” had a significant effect on infants’ first look duration (*χ*^2^(1) = 15.39, *p* = < .001, estimate = 0.40, *SE* = .10), with their looking times at identity changes being longer in the “eye contact” condition (*M* = 2078.18 ms; *SD* = 978.21 ms) compared to the “no eye contact” condition (*M* = 1416.11 ms; *SD* = 734.48 ms). In addition, we found a main effect of “identity change” (*χ*^2^(1) = 14.19, *p* = < .001, estimate = .27, *SE* = .07) and “location change” (*χ*^2^(1) = 16.04, *p* < .001, estimate = 0.29, *SE* = .07) indicating that infants’ first look duration was significantly longer compared to the baseline condition in both outcome change conditions. The interaction between first-party “eye contact and location change” did not have a significant effect (*χ*^2^(1) = 0.04, *p* = .85, estimate = −0.02, *SE* = .10). We did not find a significant effect of “running trial” (*χ*^2^(1) = 1.19, *p* = .27, estimate = −0.08, *SE* = .07), “trial within condition” (*χ*^2^(1) = 0.46, *p* = .50, estimate = 0.04, *SE* = .06), or “object position” (*χ*^2^(1) = 0.66, *p* = .42, estimate = −0.06, *SE* = .08). The results are illustrated in Figure 2B.

### Discussion

We found that 9-month-old German infants showed a selective memory bias for recognition-relevant object properties when an adult on a screen had established eye contact with them before and after looking at an object (for an overview see Table 3A). Given the non-significant full-null model comparison based on the *total looking time duration at the screen*, the results based on the individual fixed effects of the corresponding model need to be interpreted with caution. However, due to the identical analytical logic across all our statistical models, and the high consistency with our results based on the same measure in Experiment 2 and in the merged analyses including data from both experiments, we suggest the following interpretation of our results. In line with our hypotheses based on previous studies, infants tended to show an identity bias in the “eye contact” condition, that is, increased looking times at identity over location changes and compared to the baseline. The duration of infants’ total looking times at location changes did not differ from the baseline, suggesting the absence of a location bias in the “eye contact” condition. In the “no eye contact” condition, 9-month-old infants showed no longer looking times at any of the change outcomes compared to the baseline (no memory bias). This contrasts with our hypothesis based on the two result patterns found in previous studies, where infants showed a location bias (Yoon et al., 2008) or both a location bias and an identity bias (Okumura et al., 2016) in the “no eye contact” condition. Across eye contact conditions, infants’ looking times at identity change outcomes but not location change outcomes were higher in the “eye contact” compared to the “no eye contact” condition.

The results based on infants’ *first look duration at the object* revealed that, irrespective of the eye contact condition, infants showed an identity and a location bias indicated by longer looking times at identity changes and location changes compared to the baseline. In contrast to the “no eye contact” condition, however, infants’ first look duration in the “eye contact” condition was significantly longer toward identity changes compared to location changes. In line with the findings by Okumura et al. (2016), this suggests that infants were capable of encoding both identity and location-relevant object features. However, in deviation from the findings by Okumura et al. (2016), our data speaks against a *disrupted* encoding of location features, as infants’ surprise reaction toward location changes was not influenced by the presence of eye contact. Instead, the significant interaction between “eye contact and identity change” in both measures suggests an *increased* encoding of identity-relevant features. In the General Discussion, we integrate our findings from both measures and experiments and discuss them in the context of our study hypotheses.

Combining the results from both response measures, our findings from Experiment 1 align with the idea that 9-month-old infants’ memory grants identity-related object features a special role when encountering an object in a joint visual attention situation involving mutual eye contact compared to a parallel attention situation lacking interpersonal sharedness. This conceptually replicates the previous finding by Okumura et al. (2016) that gaze cues alone can bias infants’ memory toward encoding and retaining recognition-relevant object features. In the General Discussion, we provide a more detailed discussion of the similarities and differences across the result patterns based on the two dependent measures in comparison to previous studies.

To compare infants’ object encoding with a situation in which they were mere observers, we tested an additional sample of infants in a second experiment investigating infants’ object encoding in a matched third-party setting.

## EXPERIMENT 2: THIRD-PARTY CONTEXT

### Methods

The experimental design, procedure, data pre-processing and analysis procedures were identical to Experiment 1 (link to counterbalancing sheet: https://osf.io/y3mg2). We pre-registered the hypotheses, methods, procedures, and the data analysis plan for this experiment prior to data collection on the OSF (link: https://osf.io/mp9td/). The design and procedure of this experiment were approved by the same Ethics Committee as in Experiment 1.

#### Participants.

Thirty-six typically developing infants between 9 months, 1 day and 9 months, 30 days of age were included in the final sample of Experiment 2 (*n* = 18 female; *M* = 288.2 days, *SD* = 7.58 days). Data from four additional children were excluded because they did not provide the minimum amount of one valid trial per condition. Data inclusion criteria were the same as in Experiment 1. The participants were recruited from the same database.

#### Stimuli.

Analogously to the 2 × 3 design in Experiment 1, we manipulated whether two actors engaged in eye contact with one another (eye contact or no eye contact) before looking at a visible object, and the change of the object in the outcome phase (no change, identity change, location change). To manipulate the eye contact between the two actors, we used the movement of the actors’ bodies (turning toward or away from one another), the relative positioning of their bodies (face-to-face or back-to-back), and their gaze direction (eye contact or looking in opposite directions). The operationalization of “no eye contact” as was inspired by the “no eye contact – look at object” condition in the study by Thiele et al. (2021b).

**Figure 3.**
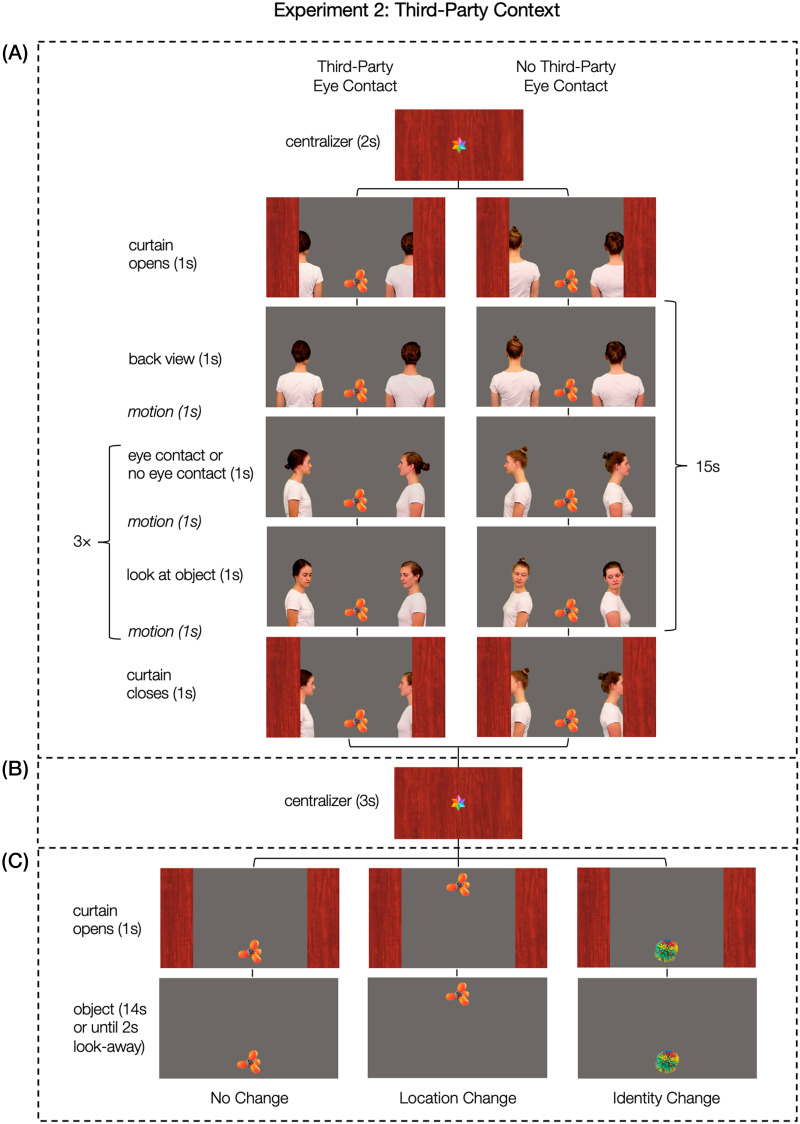
**Exemplary Sequence and Timing of one Test Trial in Experiment 2.** *Note*. Every trial consisted of (A) an action phase (15 s), (B) a delay phase (3 s), and (C) an outcome phase (15 s or until the infant looked away). Before the action and the outcome phase, an attention-getting animation (blinking star) was presented in the center of the screen. The position of the object in the action phase (top or bottom) was counterbalanced. Figure S4 in the Supplemental Materials illustrates the counterbalancing version in which the object was positioned at the top screen position in the action phase.

The videos in this experiment were edited in a way that they had the same timing and degree of motion as the videos in Experiment 1, except that they contained two actors instead of one (see Figure 3): Initially, both actors were seen in back view to make sure that the infant was not addressed in any way (1 s). Then, they turned toward or away from one another (1 s) and remained in this face-to-face or back-to-back position (1 s). Then, both actors turned their heads and gazes simultaneously in the direction of (or away from) a visible object (1 s) and remained in this position (1 s). The turn-taking between looking at each other and the object was repeated three times ending in a face-to-face (or back-to-back) sequence (stimulus examples “eye contact” condition: https://osf.io/bw53c, “no eye contact“ condition: https://osf.io/n8gpf). The size of the areas covering all possible movements of the actor’s bodies and faces was identical to the areas one actor covered in Experiment 1 (see Figure S2 in the Supplemental Materials for an illustration of the visual arrangement on screen). Like in Experiment 1, each child was presented with two trials of each condition, presented in four blocks of three trials.

Every child saw two dyads of four female actors. Two actors were the same as in the first Experiment. One dyad performed in all trials of the “eye contact” conditions and the other dyad performed in all trials of the “no eye contact” conditions. Across participants, each of the two dyads engaged equally often in eye contact as in no eye contact. Like in Experiment 1, the face-to-face dyad in the pre-test trials engaged in eye contact in the test trials, and the back-to-back dyad in the pre-test trials did not engage in eye contact in the test trials (stimulus examples pretest trials: https://osf.io/ne875).

#### Data Analysis.

Data pre-processing, trial inclusion criteria and analyses were the same as in Experiment 1. On average, infants provided 1.87 valid trials (*SD* = .34) per condition (see Table S4 in the Supplemental Materials for a detailed valid trial statistics).

### Results

**Figure 4.**
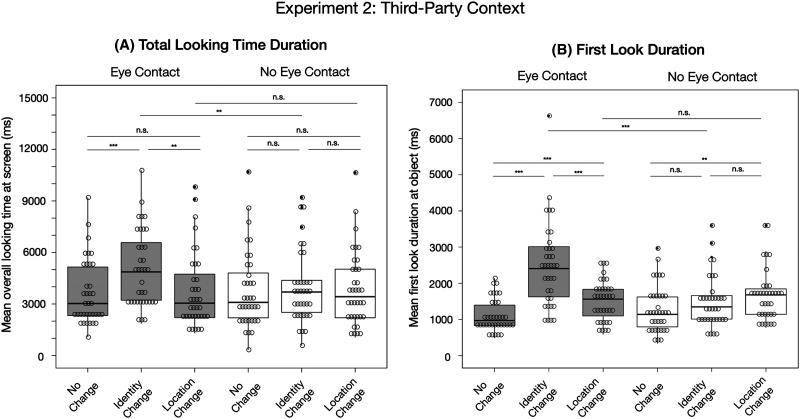
**Results from Experiment 2.** *Note*. Results are based on (A) the total looking time duration at the screen before two consecutive seconds looking away from the screen and (B) the duration of looking time at the object until looking away from the object for the first time. The significances for the pair-wise comparisons within each eye contact condition were retrieved based on the R-package *emmeans*. The significances across eye contact conditions represent the effects of the interactions between “eye contact and location change” and “eye contact and identity change” from the main model comparison.

In the following, we describe the results of our main analyses for the same measures as reported in Experiment 1. The means and standard deviations of both dependent measures are depicted in Table 2 and the significances of the post-hoc pair-wise comparisons are illustrated in Figure 4 (for more statistical details see Tables S5 and S6 in the Supplementary Materials). In addition, we report the results of a merged analysis comparing the data from Experiment 1 and 2.

**Table 2.** Means and Standard Deviations of the Two Dependent Measures (ms) for the Six Conditions in Experiment 2.

Condition	Duration of total looking time at the screen	Duration of first look at the object
Third-party eye contact	3657.03 (*1890.30*)	1102.71 (*450.25*)
No change outcome		
Third-party eye contact	5036.75 (*2225.87*)	2490.96 (*1169.52*)
Identity change outcome		
Third-party eye contact	3766.56 (*2189.52*)	1518.24 (*557.08*)
Location change outcome		
No third-party eye contact	3795.79 (*2248.37*)	1282.42 (*639.89*)
No change outcome		
No third-party eye contact	3985.07 (*2137.05*)	1482.78 (*702.26*)
Identity change outcome		
No third-party eye contact	3857.24 (*2162.68*)	1696.54 (*713.34*)
Location change outcome		

#### Total Looking Time Duration.

The comparison between the full model and the reduced model revealed a significant result indicating that at least one of the interactions had an impact on infants’ total looking time duration at the screen during the outcome phase (*χ*^2^(2) = 6.43, *p* = .04, see Figure 4A). More specifically, the interaction between third-party “eye contact and identity change” had a significant effect on infants’ total looking time duration (*χ*^2^(1) = 6.40, *p* = .01, estimate = 0.30, *SE* = .12), with the looking times at identity changes being longer in the “eye contact” condition (*M* = 5036.75 ms; *SD* = 2225.87 ms) compared to the “no eye contact” condition (*M* = 3985.07 ms; *SD* = 2137.05 ms). The interaction between third-party “eye contact and location change” did not have a significant effect (*χ*^2^(1) = 1.35, *p* = .24, estimate = 0.14, *SE* = .12). We found a significant effect of “running trial” (*χ*^2^(1) = 9.22, *p* = .002, estimate = −0.22, *SE* = .07) in that infants’ looking times decreased over time. The main effects of “identity change” (*χ*^2^(1) = 0.07, *p* = .79, estimate = 0.02, *SE* = .08), “location change” (*χ*^2^(1) = 0.80, *p* = .37, estimate = −0.08, *SE* = .09), “trial within condition” (*χ*^2^(1) = 0.78, *p* = .38, estimate = 0.06, *SE* = .07) and “object position” (*χ*^2^(1) = 0.03, *p* = .86, estimate = −0.01, *SE* = .06) did not reveal a significant effect. We found the same result pattern based on infants’ total looking duration at the *object* (for more details see section S8 in the Supplementary Materials).

#### First Look Duration.

The comparison between the full model and the reduced model revealed a significant result (*χ*^2^(2) = 45.37, *p* = < .001, see Figure 4B). More specifically, the interaction between third-party “eye contact and identity change” had a significant effect on the duration of infants’ first look at the object (*χ*^2^(1) = 37.82, *p* < .001, estimate = .68, *SE* = .11), with the duration of first looks at identity change outcomes being longer in the “eye contact” condition (*M* = 2490.96 ms; *SD* = 1169.52 ms) compared to the “no eye contact” condition (*M* = 1482.78 ms; *SD* = 702.26 ms). In addition, we found a main effect of “eye contact” (*χ*^2^(1) = 4.33, *p* = .04, estimate = −.17, *SE* = .08) in that the looking times at the object appeared significantly longer following the “eye contact” condition compared to the “no eye contact” condition. However, this effect seemed to be mainly driven by the interaction between “eye contact and identity change”, since the estimates of the main effect and the interaction pointed in opposite directions. In addition, we found a significant main effect of “location change” (*χ*^2^(1) = 9.74, *p* = .001, estimate = .24, *SE* = .08), indicating that infants’ first look duration toward location changes was higher compared to no changes, irrespective of the communicative context. The interaction between third-party “eye contact and location change” outcome did not have a significant effect (*χ*^2^(1) = 0.55, *p* = .46, estimate = 0.08, *SE* = .11). We did not find a significant main effect of “identity change” (*χ*^2^(1) = 1.50, *p* = .22, estimate = 0.09, *SE* = .08), “running trial” (*χ*^2^(1) = 2.15, *p* = .14, estimate = −0.08, *SE* = .05), “trial within condition” (*χ*^2^(1) = 0.43, *p* = .51, estimate = 0.04, *SE* = .06) and “object position” (*χ*^2^(1) = 0.76, *p* = .38, estimate = −0.06, *SE* = .07).

#### Merged Analyses Including Data from Both Experiments.

To compare the data from Experiment 1 and 2, we repeated our main analyses for both outcome measures over a merged sample including infants from both experiments (*N* = 72). In addition to the fixed effects included in the main analysis, we included experiment (1 or 2) as fixed effect. Like in the separate analyses of the two experiments, infants’ *total looking time duration at the screen* varied as a function of “eye contact and identity change” outcome (effect of the interaction: *χ*^2^(1) = 9.01, *p* = .003, estimate = 0.27, *SE* = .09). In addition, we found a significant effect of “running trial” (*χ*^2^(1) = 9.03, *p* = .003, estimate = −0.19, *SE* = .06). We did not find significant effects of the interaction between “eye contact and location change” (*χ*^2^(1) = 0.46, *p* = .50, estimate = 0.06, *SE* = .09), “experiment” (*χ*^2^(1) = 0.04, *p* = .84, estimate = 0.02, *SE* = .10), main effect of “location change” (*χ*^2^(1) = 0.25, *p* = .62, estimate = −0.03, *SE* = .06), main effect of “identity change” (*χ*^2^(1) = 0.02, *p* = .88, estimate = 0.01, *SE* = .06), “trial within condition” (*χ*^2^(1) = 0.02, *p* = .89, estimate = −0.01, *SE* = .06), or “object position” (*χ*^2^(1) = 0.35, *p* = .56, estimate = −0.03, *SE* = .05).

The merged analysis based on infants’ *first look duration at the object* also revealed a significant effect of the interaction between “eye contact and identity change” (*χ*^2^(1) = 42.54, *p* < .001, estimate = 0.52, *SE* = .08). In addition, we found significant main effects of “identity change” (*χ*^2^(1) = 13.47, *p* < .001, estimate = 0.20, *SE* = .05) and “location change” (*χ*^2^(1) = 25.28, *p* < .001, estimate = 0.28, *SE* = .06) indicating longer first look durations at both change outcomes compared to the baseline. We did not find significant effects of the interaction between “eye contact and location change” (*χ*^2^(1) = 0.01, *p* = .92, estimate = −0.01, *SE* = .08), “experiment” (*χ*^2^(1) = 2.27, *p* = .13, estimate = 0.17, *SE* = .11), “trial within condition” (*χ*^2^(1) = 1.10, *p* = 30, estimate = 0.04, *SE* = .04), “running trial” (*χ*^2^(1) = 3.38, *p* = .07, estimate = −0.07, *SE* = .04), or “object position” (*χ*^2^(1) = 1.02, *p* = .31, estimate = −0.05, *SE* = .05).

### Discussion

Previous research has shown that observed joint attention has a facilitating effect on infants’ object memory (Thiele et al., 2021b). Here, we extend this finding by showing that the memory-enhancing effect is highly specific in that it modulates *how* infants represent and encode a mutually attended object. Nine-month-old German infants showed a selective bias toward representing and memorizing recognition-relevant identity features when observing two adults attending to an object in a joint attention setting without ever looking in the direction of the infant (for an overview see Table 3B).

**Table 3.**
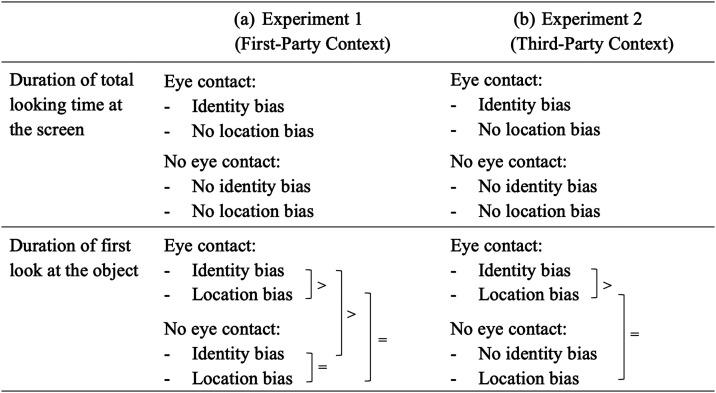
Overview of the Memory Biases in Experiment 1 and 2 for Both Dependent Measures

*Note.* A memory bias was defined as significantly longer looking times at a change outcome compared to the baseline (no change). Across both experiments and measures, infants’ looking time response toward identity changes was significantly longer in the “eye contact” compared to the “no eye contact” conditions (interaction between “eye contact and identity change”). Within the “eye contact” conditions in both experiments, the identity bias was stronger compared to the location bias (if present). Within the “no eye contact” conditions, the identity bias (if present) was equal compared to the location bias (if present). “>” indicates a stronger identity bias and “=” equally strong biases in conditions where an identity *and* a location bias were present.

Based on infants’ *total looking time duration at the screen*, infants showed the same result pattern like in Experiment 1. In the eye-contact condition they showed an identity bias, that is, increased looking times at identity over location changes and compared to the baseline (see also Okumura et al., 2016; Yoon et al., 2008). The duration of infants’ looking times at location changes did not differ from the baseline, suggesting the absence of a location bias in the “eye contact” condition. In the “no eye contact” condition, infants showed no longer looking times at any of the change outcomes compared to the baseline (no memory bias). Comparing across eye contact conditions, infants’ looking times at identity change outcomes but not at location change outcomes was higher in the “eye contact” compared to the “no eye contact” condition.

Like in Experiment 1, the results based on infants’ *first look duration at the object* revealed both a location bias and identity bias in the “eye contact” condition, with significantly longer first look durations toward identity changes compared to location changes. In contrast to Experiment 1, however, infants in the “no eye contact” condition showed only a location bias and no identity bias, similar to the result pattern in the study by Yoon et al. (2008). Across eye contact conditions, infants’ looking times at identity change outcomes but not location change outcomes were higher in the “eye contact” compared to the “no eye contact” condition.

In summary, our results suggest that the identity bias in joint attention contexts goes beyond situations in which infants are directly addressed. Additional analyses over a merged sample including participants from both experiments suggested that infants’ selective memory bias for identity-relevant information did not differ significantly between a third-party observational context and a first-party setting.

## GENERAL DISCUSSION

Previous research has shown that joint visual attention to an object with another person biases 9-month-old infants to encode qualitatively different object properties compared to a parallel attention situation lacking interpersonal sharedness (Okumura et al., 2016). The present study replicates this finding, supporting the existence of a selective memory bias in first-party joint attention contexts. In Experiment 1, 9-month-old German infants showed a selective bias toward processing and retaining identity-related object features when seeing an adult addressing the infant through direct gaze (eye contact) before and after looking at an object. In contrast, infants did not show such a selective memory bias when seeing an adult looking to the side (no eye contact) before and after shifting their gaze to an object. Moreover, this study provides first evidence that the selective memory bias is more flexible than previously shown. Crucially, in Experiment 2, we extended previous findings by showing the same result pattern in a matched third-party design, during which infants observed two adults establishing eye contact (or no eye contact) before alternating their gaze between an object and their partner without ever looking in the direction of the infant.

Tying our results back to our study hypotheses, our findings in the “eye contact” conditions of both experiments were in line with our predictions: Despite smaller differences across both experiments and measures, infants consistently showed an identity bias, that is, increased looking times at identity changes compared to the baseline and compared to location changes (in line with Okumura et al., 2016; Yoon et al., 2008). In the “no eye contact” conditions, our findings were less consistent with our initial assumptions. Based on two previous studies, we had hypothesized that two result patterns would indicate a communication-induced memory bias: Infants should either show a location bias (Yoon et al., 2008) or a location *and* an identity bias with no difference between the change outcome conditions (Okumura et al., 2016). While our results based on the first look measure in Experiment 1 aligned with Okumura et al.’s findings, our results based on the same measure in Experiment 2 resembled Yoon et al.’s findings. When using infants’ total looking time as the dependent measure, we did not find any memory bias in the “no eye contact” conditions across both experiments.

It is important to note that, even though all three studies (Okumura et al., 2016; Yoon et al., 2008; this study) relied on the same experimental paradigm, there were several deviations in methodological details. For example, how the scene was occluded during the delay phase, whether the stimuli were live or recorded, whether the location change of the object conformed to gravity or not, or whether gestures and language were involved in addition to gaze cues (for related discussions, see Silverstein et al., 2019). The fact, that three studies with varying methodological details revealed three different result patterns regarding infants’ memory biases in situations lacking communication, makes it difficult to derive conclusions about infants’ information processing in non-communicative social learning settings. We speculate that, in contrast to communicative situations, infants from the tested socialization contexts may encounter more diversity within non-communicative social learning situations (e.g., regarding a model’s actions) given that social learning settings in these contexts are structured by typical patterns of infant-directed communication. Following this assumption, it would be possible that a variety of mechanisms organizes infants’ object memory in non-communicative situations, depending on the social cues present.

When comparing the results from the communicative and non-communicative context across all three studies, we conclude that the identity bias within communicative contexts is a rather robust finding that seems to generalize across different versions of the paradigm. Importantly, despite deviating results in the non-communicative conditions, all three studies showed that infants’ object perception and memory assigned special value to identity-relevant information when encountering a novel object within a communicative social interaction involving joint attention.

### Comparing Total Looking Time Duration and First Look Duration Measure

We relied on two measures of infants’ violation-of-expectation response: the time infants spend looking at an outcome until looking away from it for two consecutive seconds (total looking time duration, main measure in study by Okumura et al., 2016) and until looking away from it for the first time (first look duration, main measure in study by Yoon et al., 2008). We included both measures to increase the comparability with results from previous studies (see also Silverstein et al., 2019). According to Yoon et al. (2008) “[…] measuring the duration of the first look before any looks away should be more sensitive to perceptual change and more interpretable as reflecting change blindness than total looking times.” (p. 13694).

Common to our results based on both measures was that, in both experiments, infants’ looking time durations were consistently longest compared to all other conditions when seeing an identity change following a joint attention scene involving eye contact. When comparing the result patterns in more detail, we found some smaller differences appearing systematically across both experiments. This deviates from previous studies using manual coding procedures, where infants’ first look duration was overall shorter compared to their total looking time, but the condition differences were similar across both measures (Silverstein et al., 2019; Yoon et al., 2008). In our study, the selective memory bias based on infants’ total looking time duration unfolded in longer looking times at identity changes compared to location changes and no changes in only the “eye contact” conditions of both experiments, and no surprise response toward location changes in any of the conditions. Based on infants’ first look duration, infants in both experiments looked longer at all change outcomes compared to the baseline (irrespective of the eye contact condition), but their first look duration was longest when seeing an identity change following a joint attention scene involving eye contact (for an overview see Table 3).

One explanation for the differences between the two measures lays in conceptual details of the processing approaches we applied to determine the timepoint of the respective look-away behaviors. When coding looking behaviors manually based on video recordings of an infant’s face, the moment of looking away is explicitly visible and consistent across both measures. In plain text eye-tracking data, however, this information must be inferred indirectly. To do so, we preregistered a data processing approach aiming at detecting the two look-away behaviors within eye-tracking data, without relying on any visual information of infants’ eye movements or head turn behaviors (see section S6 in the Supplementary Materials for more details). Additional analyses based on infants’ total looking time at the *object* ruled out that differences in AOI size were responsible for the deviations across the two measures. A more plausible explanation relates to the ways in which we inferred the timepoint ending a measurement: To determine the moment when an infant looked away for two consecutive seconds, we monitored the time intervals between consecutive screen fixations. In contrast, the first look measure ended when only one gaze sample outside the object AOI was detected (or when the gaze shift latency criterion applied). Accordingly, the first look duration represented a more conservative and possibly more sensitive indicator of infants’ violation-of-expectation response—compared to our total looking time measure and to both manually coded measures used in previous studies.

Our study provides an example that eye-tracking can be successfully applied to retrieve looking time measures with look-away based criteria. It furthermore lends support to the idea that the richness and high resolution of eye-tracking data entails the potential of providing a more sensitive insight into infants’ VoE response. In section S4 in the Supplementary Materials, we discuss the chances and limitations of our approach in more detail, considering our results from exploratory analyses based on Silverstein et al.’s (2019) eye-tracking and manually coded data. An important future avenue will be to investigate and compare the psychological drivers of infants’ first look duration and their total looking time response in this paradigm. The finding in previous studies, that the two measures produced similar results, points towards the possibility that both behaviors represent the same underlying phenomenon. Another possibility would be that the two measures are representing something different, such as different representational levels of object memory. Which of these alternatives apply may be furthermore influenced by methodological details of the paradigm. For example, the occlusion of the scene during the delay phase (completely in our study and Okumura et al., 2016; partially in Yoon et al., 2008) affected whether a location change was theoretically possible. This, in turn, may have influenced what was measured in these studies (e.g., visual recognition memory vs. encoding features of a 3D object). Furthermore, deviations in the occlusion event may have contributed to the overall weaker response toward location changes in our study.

### Possible Mechanisms Underlying Infants’ Selective Memory Bias

In previous studies, two potential mechanisms have been discussed as underlying drivers of infants’ selective memory bias in first-party communicative or joint visual attention settings. First, Yoon et al. (2008) proposed a resource-saving mechanism according to which infants use their limited resources selectively, investing them into retaining object properties that are most relevant within a given social situation. Okumura et al. (2016) proposed that the presence of eye contact *disrupts* the processing of location information—not due to lacking memory resources, but instead because spatial-temporal information is context-specific and, thus, less relevant for future recognition and learning (see also Marno et al., 2014, 2016). Our findings raise a third possibility, namely that the presence of interpersonal sharedness expressed via eye contact *enhances* the processing of identity-relevant information. Across both experiments and dependent measures, infants’ looking response to identity changes were longest in the “eye contact” conditions—compared with the other two outcomes within the “eye contact” condition and in contrast to infants’ response to all outcomes in the “no eye contact” conditions. In combination, these findings suggest that the selective identity bias may be expressed in more than one distinct result pattern—on the one hand, depending on methodological details and, on the other hand, influenced by characteristics of the social situation depicted in the stimuli. Communicative and non-communicative social contexts can involve a multitude of diverse social cues influencing infants’ object representations and, in turn, their looking time response.

Originally, Yoon et al. (2008) introduced the communication-induced identity bias as an early emerging psychological mechanism guiding infants’ learning from others in infant-directed pedagogical interactions. In our study, in contrast, we removed any pedagogical component from the stimuli—none of the actors was leading the interaction in any kind of way. Instead, like Okumura et al. (2016), we systematically manipulated interpersonal sharedness expressed via joint visual attention as a learning facilitating mechanism. Together with the study by Okumura et al. (2016), we show that the effect of joint visual attention on infants’ object representations generalizes across screen-based and live-interactive study procedures and, importantly for the focus of this study, across self-experienced and observed interaction contexts.

It remains an open question is *how* joint attention influences infants’ object encoding. One possibility we explored in our additional analyses is that joint attention to an object enhances infants’ own overt attention to the object (as also discussed in Silverstein et al., 2019). However, as we describe in more detail in sections S5 and S8 in the Supplemental Materials, we did not find evidence for this explanation. Another possibility we explored is that infants’ attention to the *actors* may predict their identity bias. This was based on a previous word learning study showing that variation in infants’ *social* attention modulated their learning outcomes (Shneidman et al., 2009). In the study, 20-month-old US American toddlers were introduced to a novel object label in either a direct interaction condition or an overhearing condition in which an experimenter addressed another adult. In the overhearing condition, children allocated more attention to the people compared to the object, and their looking time at the actors’ faces predicted their subsequent learning outcomes. The authors theorized that, when observing third-party interactions, children may search for behavioral cues indicating others’ attentional focus and that a successful search would result in successful learning. Alluding to this idea, our exploratory analyses revealed that infants in Experiment 2 looked longer at the actors’ faces in the third-party “eye contact” condition compared to the “no eye contact” condition (section S8 in the Supplemental Materials). Based on the current data, however, it remains unclear whether this was indeed caused by social motives, such as seeking of social cues or signs of interpersonal sharedness. Additional research is needed to investigate infants’ attention with a more fine-grained approach, including eye-tracking measures with higher temporal resolution (e.g., scan paths) or paradigms testing covert visual attention (for a study example with adults, see Böckler et al., 2016). This would furthermore allow to explore the possibility that infants’ attention in joint attention interactions might be qualitatively different from parallel attention situations, but not quantifiable in infants’ overt attention distribution (see also Moll & Tomasello, 2007).

### Implications for Our Understanding of Social Learning in Infancy

This study contributes to a growing body of literature emphasizing that preverbal infants can learn from others outside directed pedagogical interactions. We tested infants in urban Germany, a context where children typically experience high levels of child-directed interactions. Our finding that “even” in such a context infants showed a selective memory bias during observed joint attention, raises the possibility that the effect emerges possibly independently of the cultural context in which a child grows up. To investigate this possibility systematically, cross-cultural studies are needed. Most of what we know about infants’ observational learning in places where observation is valued, stems from ethnographic descriptions of the infant’s daily interaction experience and learning environment. Studies investigating learning *competencies* in these contexts have focused on children from around two years onwards, revealing cross-cultural variation in seeking observational learning opportunities, general attention strategies during the observation, and learning outcomes (Chavajay & Rogoff, 1999; Correa-Chávez & Rogoff, 2009; Kardan et al., 2017; Shneidman, Gaskins, & Woodward, 2016; Shneidman, Gweon et al., 2016). Studies are needed investigating when in ontogeny these differences emerge and what regularities within the child’s social experience may explain the cross-cultural variation. Possible influential factors could be socialization goals, the number of social partners providing care, parenting practices and interaction styles, and how much responsibility infants are given for organizing their attention and learning (Barnett et al., 2022; Keller, 2018; Rogoff, 1993). Moreover, to gain a more fine-grained picture of the relevance of the two learning paths within and across cultures, another interesting future avenue would be to investigate the infant’s learning *preferences*. It would be, for example, possible that infants in the here tested population can learn from first- and third-party joint attention interactions—but that they would prefer first-party interactions if they were given the choice.

In the first-party interaction literature, a growing body of literature has emphasized the infant’s active role in learning and acquiring cultural specific information from others (Begus et al., 2014; Begus & Southgate, 2018; Crivello et al., 2018; Kovács et al., 2014). In cultures where social input is predominantly observational, researchers have emphasized that this also applies to observation-based forms of learning (Gaskins & Paradise, 2010; Rogoff et al., 2003). In addition, there is some evidence that also in cultures where child-directed interactions are valued as primary social learning context, infants take an active role in the observational learning process. For example, German and Swedish infants between 7 and 13 months of age increasingly orient their attention to third-party face-to-face interactions (Galazka et al., 2014; Handl et al., 2013; Thiele et al., 2021a) and 13-month-olds are intrinsically motivated to seek out situations in which they can observe third-party interactions (Thiele, Hepach, Michel, Gredebäck et al., 2021). The present study adds to another line of research showing that infants furthermore organize their attention and referential learning during the observation situation (Elsner et al., 2014; Tatone et al., 2021; Thorgrimsson et al., 2014). Without any external guidance, 9-month-old German infants recognized interpersonal sharedness between third parties and used it as a relevant dimension when processing and memorizing a novel object.

This study focused on a gaze-based definition of joint attention. To conceptualize the phenomenon more inclusively, studies are needed broadening this definition toward including *physical* cues of interpersonal sharedness. This would be particularly important when studying the ontogeny of social learning across different cultures (Abels, 2020; Bard et al., 2021; Botero, 2016; Little et al., 2016). Another limitation is that our stimuli were created in a very controlled way to increase the internal validity and precisely match the videos across all conditions. This came along with some artificial aspect, such as the back-to-back manipulation in the “no eye contact” condition. Based on our exploratory analyses of infants’ looking behavior during the action phase, we did not find any evidence suggesting that infants’ looking response was driven by the “weirdness” of the no eye contact manipulation (see section S8 in the Supplementary Materials). Yet, it would be an important future avenue to investigate how the learning enhancing effect of joint attention unfolds in a more naturalistic social learning environment, for example, when multiple multimodal stimuli and learning opportunities compete for attention (Osborne-Crowley, 2020). In this context, it would be furthermore important to examine how characteristics of the observed people, such as their familiarity, competence, expertise, or age contribute to infants’ learning and whether this compares to features influencing infants’ learning in self-experienced interactions (Shimpi et al., 2013; Soley & Sebastian-Galles, 2020; Stenberg, 2020). In addition, future studies need to compare infants’ object encoding in observed joint attention contexts with a non-social attention-grabbing situation to tease apart the influence of general attention mechanisms and to investigate the specificity of the *social* learning response (Okumura et al., 2020; Szufnarowska et al., 2014).

## CONCLUSIONS

This study reaffirms that preverbal infants process objects differently when encountering them within such an interaction. Moreover, crucially, the study demonstrates that infants learn in the same way when observing third-party interactions, even within a socialization context where direct pedagogy represents the predominant social learning context. Our results suggest that infants in the first year of life use interpersonal sharedness to identify what and when to learn from others—not just when they are addressed directly, but all the time.

## ACKNOWLEDGMENTS

We thank all the families who participated in the study; Luke Maurits for statistical support and advice; Yujin Kim for leading the testing sessions; Katharina Haberl for study coordination; Jana Jurkat for helping with setting up the testing environment and for supporting with participant recruitment; Sarah DeTroy, Sarah Peoples, Marie Padberg, and Julia Prein for acting in the video stimuli; Priya Silverstein and colleagues (2019) for sharing their eye-tracking data with us for exploratory, supplementary analyses; as well as three reviewers for their valuable and constructive feedback that helped us improving our manuscript.

## FUNDING INFORMATION

The conduct of the research, preparation of the article, and publication of this article was funded by Max Planck Society for the Advancement of Science, a noncommercial, publicly financed scientific organization. In addition, it was supported by the internal budget of the Department of Early Child Development and Culture at Leipzig University (no grant number).

## AUTHOR CONTRIBUTIONS

Maleen Thiele: Conceptualization; Data curation; Formal analysis; Investigation; Methodology; Project administration; Supervision; Validation; Visualization; Writing—Original draft; Writing—Review & editing. Steven Kalinke: Data curation; Methodology; Software; Validation. Christine Michel: Conceptualization; Methodology; Writing—Review & editing. Daniel Haun: Conceptualization; Funding acquisition; Resources; Writing—Review & editing.

## DATA AVAILABILITY STATEMENT

The preregistrations, video examples, eye-tracking raw data, and R scripts for preprocessing and analyzing the data are available on the Open Science Framework (Experiment 1: https://osf.io/t4yqj, Experiment 2: https://osf.io/mp9td/).
