# Supplementary material for: Direct and Observed Joint Attention Modulate 9-Month-Old Infants’ Object Encoding
Source: Open Mind (Camb). 2023 Nov 27;7:917–46. doi: 10.1162/opmi_a_00114 (PMC10695677; doi:10.1162/opmi_a_00114)
Supplement: Supplementary file 1 [file opmi-07-917-s001.docx]

Direct and observed joint attention modulate 9-month-old infants’ object encoding

# Supplementary Information

**Table of contents**

S1 – A Priori Power Analysis

S2 – Supplementary Information Stimuli Creation

S3 – Supplementary Information Fixation Filter

S4 – Comparability Between Eye-Tracking and Manual Coding

S5 – Supplementary Analyses Experiment 1

S6 – Supplementary Information First Look Measure

S7 – Supplementary Information Statistical Models

S8 – Supplementary Analyses Experiment 2

S9 – Piloting Phase

References

Supplementary Figures

**Figure S1.** Areas of interest (AOIs) during the encoding phase of Experiment 1.

**Figure S2.** Areas of interest (AOIs) during the encoding phase of Experiment 2.

**Figure S3.** Exemplary sequence and timing of one test trial of the violation of expectation task in Experiment 1 illustrating a counterbalancing version in which the object was positioned at the upper screen position during the action phase.

**Figure S4.** Exemplary sequence and timing of one test trial of the violation of expectation task in Experiment 2 illustrating a counterbalancing version in which the object was positioned at the upper screen position during the action phase.

**Figure S5.** Illustration of the results of the power analysis based on 1000 simulated datasets.

**Figure S6.** Results from Experiment 1 based on the total looking duration at the object before two consecutive seconds looking away from the object.

**Figure S7.** Results from Experiment 2 based on the total looking duration at the object before two consecutive seconds looking away from the object.

**Figure S8.** Mean duration of total looking times and first look durations for each outcome grouped by communicative context based on the data by Silverstein et al. (2019).

**Figure S9.** Scatterplots with individual data points representing a merged sample of all participants included in Experiment 1 and 2 (total *N* = 41) in the study by Silverstein et al. (2019).

Supplementary Tables

**Table S1.** Valid trial statistics for the six conditions in Experiment 1.

**Table S2.** Results from post-hoc pair-wise comparisons of the total looking time duration at the screen within each eye contact condition between the outcome conditions of Experiment 1.

**Table S3.** Results from post-hoc pair-wise comparisons of the first look duration at the object within each eye contact condition between the outcome conditions of Experiment 1.

**Table S4.** Valid trial statistics for the six conditions in Experiment 2.

**Table S5.** Results from post-hoc pair-wise comparisons of the total looking time duration at the screen within each eye contact condition between the outcome conditions of Experiment 2.

**Table S6.** Results from post-hoc pair-wise comparisons of the first look duration at the object within each eye contact condition between the outcome conditions of Experiment 2.

**Table S7.** Means and standard deviations (ms) of the total looking time at the object in comparison to the two dependent measures reported in the main manuscript for the six conditions in Experiment 1.

**Table S8.** Results from post-hoc pair-wise comparisons of the total looking time duration at the object within each eye contact condition between the outcome conditions of Experiment 1.

**Table S9.** Means and standard deviations (ms) for looking times during the action phase in Experiment 1.

**Table S10.** Means and standard deviations (ms) for the identity bias for all three outcome measures in Experiment 1.

**Table S11.** Means and standard deviations (ms) of the total looking time at the object in comparison to the two dependent measures reported in the main manuscript for the six conditions in Experiment 2.

**Table S12.** Results from post-hoc pair-wise comparisons of the total looking time duration at the object within each eye contact condition between the outcome conditions of Experiment 2.

**Table S13.** Means and standard deviations (ms) for looking times during the action phase in Experiment 2.

**Table S14.** Means and standard deviations (ms) for the identity bias for all three outcome measures in Experiment 2.

Video examples, eye-tracking raw data and scripts for pre-processing and analyzing the data are openly assessable on the Open Science Framework:

Experiment 1: <https://osf.io/t4yqj/?view_only=43212146399a40619e0f7baed36473a7>

Experiment 2: <https://osf.io/mp9td/?view_only=0bc8ef1d17514746a42592e981916f97>

# S1 – A Priori Power Analysis

To account for all counterbalancing factors in the study, we planned to test *N* = 36 infants. To determine whether this sample size was sufficient to detect the expected effect size, we ran a simulation-based a priori power analysis based on the raw data by Okumura, Kobayashi, & Itakura (2016). We assumed that this data (*N* = 28 infants) would represent a valid basis for estimating the effect sizes in our study, because (a) we manipulated the same factors as in the study by Okumura et al. (2016) (*social context:* joint attention, no joint attention; *outcome:* no change, identity change, location change) and (b) used conceptually the same dependent measure (looking time at the screen during the outcome phase but in contrast to Okumura et al. (2016) measured via eye-tracking).

## Model Description

We compared the fit of two generalized linear mixed models (GLMMs) using ration tests: a full model and a reduced model. Both models included the same fixed effect factors as the corresponding models planned in the main analysis (see section “Analyses” in the preregistration). To account for the repeated measure structure resulting from the within-subjects design, we included subject as random intercept in the model.

The models in the power analysis deviated in two regards from the models planned in the main analysis: First, we did not include the control variable “trial within condition” (1 to 2) in the power analysis. The reason for this deviation was that in previous similar studies (Okumura et al., 2016; Silverstein et al., 2019; Yoon et al., 2008) infants were presented with one single trial per condition, making it difficult to estimate the effect of the repeated measure. Second, we did not include the full random effect structure in the models as planned in the main analysis.

***Dependent variable.*** We used the total looking duration to the screen after object reveal (ms) as the main dependent measure (gamma-distributed). We decided to use a non-normal distribution based on the assumption that looking time responses typically follow a right-skewed distribution (Csibra, Hernik, Mascaro, Tatone, & Lengyel, 2016; see also Lo & Andrews, 2015). To determine which non-normal distribution would be most appropriate for our expected looking time response measure (gamma or lognormal), we fitted maximum likelihood estimates of lognormal and gamma distributions to the raw data in each of the six conditions by Okumura et al. (2016) using the R-package “fitdistrplus”. The comparison revealed that the gamma distribution had a higher likelihood in five out of the six conditions and would therefore provide a better overall fit to the Okumura data compared to a lognormal distribution. This is in line with a recent infant looking time study by Simkovic & Träuble (2021) showing that compared to Weibull, lognormal and normal distribution, the gamma distribution shows the best fit in terms of log-likelihood and mean absolute error and the best predictive performance. In the simulated data set, we set the rate parameter of the gamma distribution to the default value of 1 and varied the shape parameter (equal to the distribution mean with rate=1) across conditions.

***Fixed factors (reduced model).*** The reduced model included eye contact and outcome as main fixed effect variables. Eye contact was included as a binary variable (eye contact: 1/0) and outcome was included as two binary dummy variables (location change: 1/0, identity change: 1/0, no change: intercept). In addition, we included fixed effect control variables for object position (top and bottom) as well as for running trial number, to account for possible overall habituation effects to the task (1 to 12, irrespective of condition).

***Fixed factors (full model).*** In addition to the fixed effects included in the reduced model, the full model included two interactions: the interaction between eye contact (1/0) and location change (1/0), and the interaction between eye contact (1/0) and identity change (1/0).

## Calculating the Expected Model Estimates

***Estimating the model parameters of the main fixed effects.*** To estimate the model parameters of the main fixed effects in our data simulation, we used the differences between the condition means of the looking time data by Okumura et al. (2016). For each simulation, a new value of each effect was sampled from a normal distribution centered on the mean differences with a standard deviation equal to one quarter of the mean. This means that in approximately 95% of simulations the effects were between 50% and 150% of those reported by Okumura, thereby incorporating some uncertainty about the true effect sizes. The simulated model estimates could vary one quarter of the mean differences around the means (+/- 2 SDs).

***Estimating the model parameters of the control fixed effects.*** To estimate possible habituation effects (i.e., effects of running trial number), we used the looking time data and valid trial statistics from previous studies using third-party interaction stimuli with similar attractiveness in terms of interactive content, timing, and visual features (Thiele, Hepach, Michel, Gredebäck, & Haun, 2021; Thiele, Hepach, Michel, & Haun, 2021b). We assumed that these studies would represent a valid reference, following the assumption that overall trial effects would result from an overall decreasing interest in the videos. Informed by this data, we assumed a small negative overall trial effect in the power analysis. Moreover, we assumed that infants would provide valid data in all 12 possible trials with a probability varying between 70% and 90% with each simulation.

We estimated a small effect of object position (top and bottom) in our simulated dataset, informed by the data from Okumura et al. (2016). In contrast to the vertically displaced object positions in our study (top/bottom), the object positions in the study by Okumura et al. (2016) were horizontally displaced (right/left), at a noticeable distance of 60 cm. Like in our study, the position of the object during the action phase was counterbalanced across conditions. The authors did not control for object position statistically, but the overall result pattern in their study speaks against a systematic effect of object position.

## Specifying the Expected Effect Size

To estimate the expected effect size, we calculated the difference of the means of the Okumura data between the critical two interactions (Interaction 1: eye contact × location change; Interaction 2: eye contact × identity change). We used the maximum difference as a reference point (2730 ms) to evaluate the outcome of the power analysis.

## Results

The power analyses based on 1000 simulations indicated that, in order to have an 80% chance that the full-null model comparison reveals a statistically significant effect (alpha level .05), the maximum of the two interactions needs to be at least ≈1500 ms (see Figure S5). Since in the observed data by Okumura et al. the expected maximum was above this threshold (2730 ms), we concluded that a sample size of 36 infants would provide sufficient power to detect an effect of the expected effect size.

## S2 – Supplementary Information Stimuli Creation

We used Adobe Premiere Pro for cutting and editing the videos. All actors were filmed individually in front of a green screen. This allowed us to (a) control for color and luminance differences between and within videos and (b) position the actors and flexibly and accurately in such a way that their overall motions were centered around the same vertical axis across all stimuli. While filming, metronome clicks were played at 120 bpm to ensure consistent timing of actions between actors and trials. When the action timing of an actor was not accurate, we corrected it frame-by-frame during post-editing in Adobe Premiere Pro. We used Adobe Premiere’s Ultra Key tool to isolate the actors from the background and replace it with an even-colored, grey background layer. Figure S1 (Experiment 1) and Figure S2 (Experiment 2) illustrate the areas that all actors’ movements covered across all conditions.

The most salient visual difference between our video stimuli and the live interactive study setting by Okumura et al. (2016) was that the object positions alternated on the vertical central axis of the screen in our stimuli (top or bottom) instead of on the horizontal axis in the study by Okumura et al. (right or left, with a noticeable distance of 60 cm). To account for possible surprise reactions elicited by the vertical change, we familiarized each infant with both screen positions during the pretest trials and included object position as a control variable in our statistical models. We did not find an effect of object position in any of the model comparisons.

# S3 – Supplementary Information Fixation Filter

To define fixations, we used the Tobii Velocity-Threshold Identification (I-VT) fixation filter with the following default parameter values: a velocity and distance threshold of 30° per second, no noise reduction, a maximum time between fixations of 75 ms, a maximum angle between fixations of 0.5°, a minimum fixation duration of 60 ms, and an interpolated of missing data for data segments below 75 ms. More details are provided in this document provided by Tobii Pro (Stockholm, Sweden): <https://www.tobiipro.com/siteassets/tobii-pro/learn-and-support/analyze/how-do-we-classify-eye-movements/determining-the-tobii-pro-i-vt-fixation-filters-default-values.pdf>

# S4 – Comparability Between Eye-Tracking and Manual Coding

One major methodological difference between the current study and previous related studies is that we used eye-tracking to extract the dependent measures. In contrast, Yoon et al. (2008), Okumura et al. (2016), and Silverstein et al. (2019) coded the infant’s looking times manually based on video recordings of the infant’s faces. The main reason for choosing a manual coding approach in these studies was the reliance on look-away behaviors as a behavioral indicator of infants’ violation-of-expectation response. The first look ended when an infant looked away from the screen for the first time, and the total looking duration measure ended when an infant looked away from the screen for two consecutive seconds. Implementing look-away-based criteria like these with eye-tracking data is challenging as no explicit information about head turns or look-away behaviors are provided in the plain text data. When an individual looks away from the screen, this is not indicated as a “look-away” in the data, but instead it appears as a missing value (NA), which is indistinguishable from missing values caused by other reasons such as recording errors or blinks. One methodological objective of the current study was to overcome this challenge. As described in the main manuscript, we developed a preregistered data processing approach aiming at (a) increasing the comparability with previously used manual coding approaches while (b) using the benefits of automated eye-tracking, such as its higher spatial resolution and the possibility to extract fixations.

In addition to the preregistered analysis plan, we explored the comparability between the data retrieved from our automated eye-tracking approach and data retrieved through manual coding. Since we did not record the infant’s faces in our study, we could not compare the two approaches based on our own data. Instead, we used the eye-tracking raw data by Silverstein et al. (2019) and compared it with their manually coded data. The authors originally recorded the eye-tracking to analyze the infant’s looking times and gaze patterns during the *action* phase. To extract the infant’s looking-time response in the *outcome* phase, the authors relied on manual coding. Nevertheless, the eye-tracker recoded the infant’s eye gaze throughout the experiment, including the outcome phase.

To compare the outcome data retrieved from our eye-tracking approach with the manually coded data by Silverstein et al. (2019), we extracted the same measures from the eye-tracking raw data that we used in our study (total looking time at the screen, total looking time at the object, first look duration at the object) and correlated them with the respective measures Silverstein et al. (2019) retrieved via manual coding.

## Participants

Out of the *N* = 48 infants included in the final sample in the study by Silverstein et al. (2019) (Experiment 1: *n* = 24, Experiment 2: *n* = 24), we included *N* = 41 infants in our analyses (Experiment 1: *n* = 21, Experiment 2: *n* = 20). In Experiment 1, three participants were excluded from the sample due to erroneous file naming in the eye-tracking raw data. In Experiment 2, four participants were excluded because of “bad eye-tracking data” according to a note in the openly available participant sheet (*n* = 3) or because they had seen two of the conditions twice according to the information provided in the eye-tracking raw data (*n* = 1).

## Data and Coding

Most relevant data and information including the eye-tracking raw data, the manually coded data, as well as tables with participant descriptions and counterbalancing information were openly accessible on the OSF in a project by Silverstein et al. (2019). In addition, the authors were very supportive in sharing their eye-tracking raw data with us upon request and uploading this data in their OSF repository (<https://osf.io/77gpt/>).

The eye-tracking data were recorded with the same eye-tracking hardware (Tobii TX120) and software (Tobii Studio, Version 3.3.1). The same filter was used to define gaze events and group individual gaze points into fixations (Tobii I-VT fixation filter with default parameter values).

In a first step, we adjusted our R script for pre-processing the eye-tracking raw data to the specifications of the data by Silverstein et al. (2019). This included a mapping of the video names in the eye-tracking raw data with the information provided in the counterbalancing table to determine for each video (a) which social context was being displayed (communicative or non-communicative), (b) what kind of outcome was being shown (identity change, location change, no change), and (c) at what screen position the object appeared in the outcome phase. The main conceptual difference between the eye-tracking data by Silverstein et al. (2019) and the eye-tracking data in our study was that every trial consisted of one single video in the Silverstein et al. (2019) data, including the action phase, the delay phase, and the outcome phase. In our study, in contrast, each trial consisted of two videos: one video containing the inter-trial attention-getter, the action phase, the delay phase, and the attention-getter before the outcome phase; and the second video containing only the outcome phase, beginning with the curtain opening. Using a separate video for the outcome phase was crucial for our automated data extraction approach because it ensured that the beginning of the outcome phase was marked with an own timestamp in the eye-tracking raw data that was not influenced by issues such as graphical or processing lags during video presentation. This was important for our time-sensitive processing algorithms, which relied on the exact time-point of the curtain opening in the outcome phase.

To determine the starting timepoint of the outcome phase in the data by Silverstein et al. (2019) as accurately as possible, we used some of the authors’ openly accessible stimulus examples and determined the exact video frame when the curtain started to open. In Experiment 1, the curtain opened 20433 ms after the video had started and in Experiment 2 it opened after 16166 ms. To find these timepoints in the eye-tracking raw data, we jumped to the recording timestamp that was close to 20433 ms (Experiment 1) and 16166 ms (Experiment 2) after video onset, assuming that the curtain must have opened around this timepoint.

Another deviation from our study was that the objects in the stimuli by Silverstein et al. (2019) were not matched regarding their shape and size. In our study, in contrast, the objects were edited in a way that each object filled an area of 360×360 pixels. If an object could not be matched into a square shape, it was stretched or compressed. This pre-editing of the objects was initially done for a previous study by Thiele, Hepach, Michel, & Haun (2021b). Section S1 in the Supplemental Information of the study by Thiele et al. (2021b) describes the detailed steps of the visual matching procedure (link to Supplemental Information: <https://osf.io/2uz97>). Especially for extracting the first look measure it was crucial that the objects were positioned at the exact same screen position and that they covered a visual area that was as consistent as possible across objects. To give an example: Following our pre-registered definition, the first look ended when a gaze sample with coordinates outside the object AOI was detected or when the latency between two consecutive object fixations was more than 3 SDs longer than the median of a child’s gaze shift latency within the object AOI (assuming that the child had looked away in this case). Even if all objects would be covered with one big AOI covering all possible objects, bigger objects would probably cause systematically longer gaze shift latencies compared to smaller objects presented in the same AOI. Moreover, it would be possible that the same distance between two consecutive fixations (in pixels) could count as a look “outside the object AOI” for a bigger object while it would not necessarily cause a leaving of the object AOI when a smaller object was displayed within the same AOI.

To control for these issues in the data by Silverstein et al. (2019), we decided to create the object AOIs rather bigger than smaller to ensure that our processing approach would not “favor” one object over another and that all objects would be treated as similar as possible by the first look duration measure. As an orientation, we used the object AOIs the authors defined for their scene analyses. To account for the inter-object variation in size and shape we made some adjustments. First, we made sure that the object AOIs had the same distance from the central vertical axis. The closest distance between the central vertical axis and object AOI was 25 pixels (right object AOI). Using this as an orientation, we positioned both the right and the left AOI 25 pixels away from the central vertical axis (i.e., 50 pixels apart from one another). Second, we ensured that the outer edges of the AOIs were equally distant from the outer edges of the screen. Here, we used the bottom edges of the original AOIs as an orientation, which aligned with the bottom edge of the screen. Analogously, we used the right border of the screen as the outer edge of the right object AOI, and the left border of the screen as the outer edge of the left object AOI. The resulting AOIs were identical in size (695×740 pixels). To set the screen AOI, we expanded the screen resolution by 1° visual angle on all sides to accommodate for inaccuracies in calibration. This approach was identical to setting the screen AOI in our study.

In the study by Silverstein et al. (2019), each child was presented with one trial per condition, that is, six trials in total. In line with our inclusion criteria, we only included trials in which the child had looked at the object for at least one fixation in the outcome phase based on the eye-tracking data. As a consequence, some children contributed less than six trials. This would have been a reason to exclude a child from the analysis in our study and in the study by Silverstein et al. (2019). However, for the purpose of the methodological validation we decided to include all trials with valid data.

## Statistical Analyses and Results

To explore the comparability between the data retrieved from our automated approach and the data retrieved via manual coding, we visually inspected the descriptive data patterns and conducted correlation analyses.

***Visual inspection of the descriptive data patterns.*** First, we plotted the manually coded looking time data by Silverstein et al. (2019) for each of the two measures (first look duration and total looking time) for both experiments. This data was also plotted in Figures 2 and 3 in the paper by Silverstein et al. (2019). However, to create an accurate basis for our comparison, we plotted the data again after excluding the participants that did not provide valid eye-tracking data. We used the openly available R code by Silverstein et al. (2019) to generate the plots. Figure S8 shows exemplary illustrations including the data of Experiment 1. In a second step, we plotted the three main dependent measures extracted by our eye-tracking approach: As analogous measures to the manually coded “total looking time”, we used the mean total looking time at the screen (Figure S8c) and the mean total looking time at the object (Figure S8d). As an analogous measure to the manually coded “first look duration”, we used the mean first look duration at the object (Figure S8e).

***Correlation analyses.*** Second, we ran correlation analyses between each eye-tracking measure and the corresponding measure extracted via manual coding. To increase the power for the correlation analysis, we combined the data from a merged sample including participants from Experiment 1 and 2. To allow for a comparison with the Experiment 1 data illustrated in Figure 2 in the main manuscript, we report the correlations for Experiment 1 in addition to the correlations based on the merged sample including participants from both Experiments. To account for the dependency of data points due to the within-subjects design, we calculated repeated-measure correlations using the R-package *rmcorr* version 0.5.4 (Bakdash & Marusich, 2022). Like the Pearson correlation coefficient “r”, the repeated-measures correlation coefficient “rmcorr” is bounded by −1 to 1 and represents the strength of the linear association between two variables (Bakdash & Marusich, 2017).

Our correlation analyses revealed medium to large correlations between the manually coded total looking time measure and each of the two analogous eye-tracking measures. Specifically, the manually coded looking time measure correlated with the total looking time at the *screen* (Experiment 1: *n* = 21; *r*(104) = .67, *p* < .001, 95% CI [.55, .77]; merged sample of Experiment 1 and 2: *N* = 41; *r*(208) = .64, *p* < .001, 95% CI [.55, .71]) and with the total looking time at the *object* (Experiment 1: *n* = 21; *r*(72) = .63, *p* < .001, 95% CI [.46, .75]; merged sample of Experiment 1 and 2: *N* = 41; *r*(151) = .40, *p* < .001, 95% CI [.26, .52]). The manually coded first look duration measure was not significantly correlated with the first look duration at the object extracted from the eye-tracking data (Experiment 1: *n* = 21; *r*(72) = .06, *p* = .61, 95% CI [−.01, .36]; merged sample Experiment 1 and 2: *N* = 41; *r*(151) = .10, *p* = .20, 95% CI [−.06, .26]). The relations between the measures are illustrated as scatterplots in Figure S9.

## Discussion

We draw the following conclusions from our findings. First, visual inspections of the data patterns and the results from the correlation analyses suggest that the two eye tracking measures using the two-second look-away criterion were most comparable with the manually coded data in the study by Silverstein et al. (2019). The total looking time at the *screen* was most similar, but also the total looking time at the *object* revealed a statistically meaningful medium to large correlation. For our own study, this suggests that the findings relying on these measures are most comparable with the total looking time measures used in previous studies (main measure in Okumura et al., 2016, additional measure in Yoon et al., 2008).

Compared to the total looking time measures, the first look duration measure was less comparable with the first look duration measure applied in studies using manual coding. There are different reasons why this may have been the case. One possibility is that, compared to the total looking time measures, our implementation of the first look duration measure was relatively less similar to the manual coding approach, as only one gaze point outside the object AOI would end the first look. This is a much more conservative rule compared to using the time interval between consecutive fixations like in our total looking time measure. It would be possible that our first look duration measure provides a more sensitive approach to measure infants’ surprise response compared to previous manually coded first look duration measures. Future research is needed to investigate this assumption more systematically.

It needs to be pointed out that the results from our comparison between the manually coded first look duration measure and the corresponding eye-tracking-based measure should be interpreted with caution. The lack of a correlation between the two measures does not mean that the first look duration measure—as we implemented it in our processing approach—is generally not interpretable or not comparable to a manually coded measure. It rather implies difficulties in comparing our first look duration measure with the manually coded first look duration measure by Silverstein et al. (2019). A plausible explanation for the lacking correlation could be that the procedure and stimuli in the study by Silverstein et al. (2019) were in many ways not suitable to produce valid data using the sensitive criterion we implemented to determine the end of the first look in our approach. To give an example: We cannot exclude that the curtain was still closed at the calculated timepoint, for example, due to graphic lags caused by the presentation software or the PC. Nevertheless, our algorithms started to seek for the cut-off timepoint where the first look ended. When the curtain actually opened, the first gaze sample might have already left the object AOI and the first look ended according to our criterion. In other words, we cannot be certain what the child was seeing at the calculated timepoint. It is, thus, unclear whether we measured the first look duration at the object or whether we measured a random first look duration at the object area while the curtain was still closed and no object was displayed.

Another difference between the study procedure by Silverstein et al. (2019) and our procedure was that the authors did not show any attention getter in the center of the screen before the curtain opened to reveal the object in the outcome phase (see also Yoon et al., Okumura et al.). This was not necessary in these studies, as they relied on the infant’s attention to the overall screen instead of relying on precise looking behaviors toward specific screen locations. However, for our first look duration measure, this is another confounding factor as it is possible that the child was already looking at one of the object AOIs.

It needs to be pointed out that all the mentioned differences between our study and the study by Silverstein et al. (2019) should not be misinterpreted as limitations of the study by Silverstein et al. (2019). The mentioned aspects that were important to control for the data processing approach in our study were just not relevant for the aims of the study. In order to draw valid conclusions about the comparability of a first look duration extracted via our eye-tracking approach and a first look duration extracted via manual coding, future studies are needed controlling for all the specifications required for our algorithms to determine the end timepoint of a first look. Adjusting our processing approach to the data by Silverstein et al. (2019) revealed the high sensitivity of our operationalization of the first look duration measure. It furthermore emphasized the importance of considering experimental details when relying on our first look duration measure, such as closely matched object size and object positioning in the stimuli, and a stimulus presentation procedure revealing precise timestamp information about the timepoint when the curtain opens.

# S5 – Supplementary Analyses Experiment 1

## Preregistered exploratory analyses

To make use of the higher spatial precision possible with automated eye-tracking, we repeated our total looking time analysis focusing on infants’ total looking times at the *object* instead of the entire screen. We planned this analysis as an exploratory analysis in the preregistration to investigate the influence of AOI size in our data processing approach and to increase comparability with the total looking time at the screen measure.

**Total looking duration at the object.** We defined a square-shaped AOI covering the object in the outcome phase. Analogously to the total looking time measure focusing on the screen AOI, the total looking time at the *object* was defined as the cumulative length of all fixations within the object AOI, beginning at the first frame of the curtain opening and ending when the time interval between two consecutive object fixations was longer than 2000 ms or after 15 seconds elapsed. Table S2 depicts the means and standard deviations of the total looking duration at the object are depicted in comparison to the means and standard deviations of both dependent measures reported in the main manuscript.

We ran the same model comparison as for our two main measures (see main manuscript), revealing a similar result pattern. The comparison between the full model and the reduced model revealed a significant result indicating that at least one of the interactions had an impact on infants’ total looking time duration at the screen during the outcome phase (*χ^2^*(2) = 13.00, *p* = .002, see Figure S6). More specifically, the interaction between third-party eye contact and identity change outcome had a significant effect on infants’ looking time response (*χ^2^*(1) = 11.55, *p* = <.001, estimate = 0.46, *SE* = .13), with the total looking time duration at the identity change outcome being longer in the “eye contact” condition (*M* = 3765.78 ms, *SD* = 1694.58 ms) compared to the “no eye contact” condition (*M* = 2904.26 ms, *SD* = 1875.43 ms).

In addition, we found a main effect of eye contact condition in that the looking time at the object was significantly longer following the “eye contact” condition compared to the “no eye contact” condition (*χ^2^*(1) = 4.15, *p* = .04, estimate = −.22, *SE* = .11). However, this effect seemed to be mainly driven by the interaction between eye contact and identity outcome, as indicated by the estimates pointing in opposite directions. The interaction between third-party eye contact and location change outcome did not have a significant effect (*χ^2^*(1) = 0.56, *p* = .45, estimate = 0.11, *SE* = .14). We did not find a significant effect of running trial (*χ^2^*(1) = 3.49, p = .06, estimate = −0.19, *SE* = .10), trial within condition (*χ^2^*(1) = 0.10, *p* = .75, estimate = −0.03, *SE* = .08), or object position (*χ^2^*(1) = 1.39, *p* = .24, estimate = −0.09, *SE* = .08). Results from the post-hoc pair-wise comparisons in Table S3.

The results reflect the same memory biases as summarized in Table 3 in the main manuscript for the duration of infants’ total looking time at the *screen* (Experiment 1). This suggests that differences in AOI size could not explain the differences in result patterns based on the first look duration measure and the total looking time measure.

## Exploratory analyses not planned in the preregistration

We conducted the following analyses in addition to the preregistered analysis plan to explore the data further.

**Looking times in the action phase.** First, we compared whether infants’ overall attention to the videos in the action phase different between communicative and non-communicative condition. For this purpose, we fitted three generalized linear mixed models with gamma distribution: one for infants’ overall looking time to the entire screen during the action phase, one for their overall looking time to the actors’ faces, and one for their overall looking time to the object (dependent variables). We included the same fixed and random effects in all three models. As main fixed effects, we included eye contact (eye contact, no eye contact), as well as running trial and trial per condition (control fixed effects). As random effects we included subject as intercept as well as random slopes on subject for running trial, trial within condition, and eye contact. We included looking times of the entire trial sequence. The AOIs used for the analyses of the data in the action phase are illustrated in Figure S1. The significance of the individual fixed effects was based on likelihood ratio tests comparing the full models with the respective reduced models excluding the individual fixed effects using the drop1-function in R with an alpha-level of .05.

We did not find condition differences in infants’ looking times at the overall screen (*χ^2^*(1) = 0.19, *p* = .67, estimate = −.01, *SE* = .03), infants’ looking times at the object (*χ^2^*(1) = 0.27, *p* = .60, estimate = .07, *SE* = .13), and infants’ looking time at the actors’ faces (*χ^2^*(1) = 0.95, *p* = .33, estimate = −.04, *SE* = .04). The means and standard deviations of the looking times in the action phase are depicted in Table S9.

**Relation between object-related attention and identity encoding bias.** Second, we explored whether infants’ selective encoding of identity-relevant features in the eye contact condition was driven by their overt attention to the object during the action phase. For this purpose, we conducted linear mixed models for infants’ identity bias (dependent variable). We calculated the identity bias by subtracting infants’ looking time in the identity change condition by their looking time in the no change condition (Looking time response toward identity change – looking time response toward no change). We conducted three models, using infant’s total looking time at the screen, their total looking time at the object, and their first look duration at the object as a foundation to calculate the identity bias. As fixed effects, we included the total looking time at the object in the action phase (z-transformed) and eye contact (eye contact, no eye contact). To account for the dependency of the within-subjects design, we furthermore included subject as a random intercept.

We found that infants’ identity bias was not influenced by the looking time at the object in the action phase—neither when using the identity score based on the total looking time at the screen (*χ^2^*(1) = 1.14, *p* = .29, estimate = −357.9, *SE* = 323.0), nor when using the identity score based on the total looking time at the object (*χ^2^*(1) = 2.44, *p* = .12, estimate = −420.5, *SE* = 264.4) or the first look at the object (*χ^2^*(1) = 0.003, *p* = .95, estimate = −5.35, *SE* = 85.52). Analogously to the results from our main analyses, we found a significant main effect of eye contact condition in all three model including infants’ total looking time at the object and infants’ first look duration at the object, in that infants’ identity bias was significantly higher in the “eye contact” condition compared to the “no eye contact” condition. Table S10 shows the means and standard deviations of the identity bias and the location bias for all three measures in both eye contact conditions.

Taken together, we did not find any indication that infants’ overt attention distribution in the action phase of Experiment 1 differed across conditions or that it caused their selective identity bias in the third-party joint attention condition. Neither was the actors’ gazing to the object following eye contact related to infants’ own attention to the object during the action phase, nor was infants’ own attention to the object related to their identity bias in the outcome phase.

# S6 – Supplementary Information First Look Measure

One difference between our first look measure and the equivalent manually coded measure used in previous studies (Silverstein, Gliga, Westermann, & Parise, 2019; Yoon, Johnson, & Csibra, 2008) was that we focused on direct looks at the object instead of the entire screen. As we describe in the preregistration, the reason for this narrower focus on overt attentional looks at the object was that—compared to an offline manual coding procedure based on video recordings of the infant’s face—eye-tracking data does not provide explicit markers of look-away behaviors. We therefore developed a workaround allowing us to indirectly infer the moment when a child stopped looking at a stimulus.

Implementing look-away-based criteria with plain text eye-tracking data is challenging. When a child looks away from the screen, this is not indicated as a “look-away” in the data, but instead it appears as a missing value (NA), which is indistinguishable from missing values caused by other reasons such as recording errors or eye blinks. To determine the moment when an infant’s gaze has left the screen we first searched for gaze samples laying outside a target Area of Interest (AOI), following the logic that this would explicitly show that an infant’s gaze was no longer within the target AOI. If we would have used the entire *screen* as the target AOI, we would have reduced the availability of such “no-target gaze samples”. Even though remote eye trackers (including the model we used) principally record gaze data outside the screen if within the trackable range, those data are rarely recorded and relatively poorer in accuracy and precision because they lay outside the calibrated tracking range. Using the *object* AOI as the target area to determine the first look duration, the remaining screen area exclusive of the object AOI provided us with a relatively bigger trackable area—increasing the likelihood of detecting a sample outside the target AOI.

To accommodate for the remaining risk that the first look away from the object may have ended with a look-away without the outwards moving saccade being detected by the eye tracker (e.g., due to a child closing their eyes and then turning their head), we decided for an additional time criterion accounting for each individual’s saccadic latency or eye movement speed during their looks at the object AOI. Taken together, the first look ended (1) if no gaze sample was detected outside the object AOI, and (2) if two consecutive object fixations were 3 SDs longer than the median gaze shift latency of the time difference between all consecutive object fixations of an individual child. The additional gaze shift latency criterion relied on the median rather than the mean latency, as the median latency is less affected by outliers (e.g., caused by missing values) and therefore more robust compared to the mean latency.

# S7 – Supplementary Information Statistical Models

We used R software environment (R version 4.2.3, RStudio version 2023.03.0) for pre-processing and analyzing the data and for setting AOIs.

## Model Descriptions

We fitted six models for each of the two experiments: Three full models and three reduced models (one full model and corresponding reduced model for each of the three dependent variables). The general structure of all models was as follows:

***Full model*** <- glmer (Looking time ~

Eye Contact (1/0) : Identity change (1/0) + Eye Contact (1/0) : Location change (1/0)

+ Eye Contact (1/0) + Identity change (1/0) + Location change (1/0)

+ z.Trial (Run) + z.Trial (Condition) + Object position

+ (1 + z.Trial (Run) + z.Trial (Condition) + Object Position + Eye Contact || ID)

, data=overall.data, family=Gamma(link=log), control=contr*)

***Reduced model*** <- glmer (Looking time ~

Eye Contact + Identity change + Location change

+ z.Trial (Run) + z.Trial (Condition) + Object position

+ (1 + z.Trial (Run) + z.Trial (Condition) + Object Position + Eye Contact || ID)

, data=overall.data, family=Gamma(link=log), control=contr*)

*contr = optimizer “bobyqa” with a set maximum of 100000 iterations.

# Main Analysis Steps

**Fitting the models.** First, we fitted the models using the R-package *lme4* version 1.1-32 (Bates et al., 2022). Our overarching goal was to fit a full model and a reduced model that would converge to all three preregistered dependent variables (total looking time duration at the screen, total looking time duration at the object, first look duration at the object) in both experiments (12 models in total). As planned in the preregistration, we first included a full random effect structure in the models (i.e., random slopes on subject for eye contact condition, location change, identity change, the interaction between eye contact and location change, the interaction between eye contact and identity change, running trial, trial within condition, and object position). However, most of our models including this random effect structure did not converge. Out of all possible random slopes for the main fixed effects, only the random effect of eye contact on subject could be reliably estimated on an individual level for all 12 models. We conducted the following steps to address the convergence issue in our models: First, we reduced the complexity of the random effect structure by excluding the interaction terms. Second, we used the optimizer “bobyqa” with a set maximum of 100000 iterations, and third, we z-transformed the covariates running trial and trial within condition.

In Experiment 2, the model fitting for the first look measure produced a boundary fit warning (full model) and a convergence warning (reduced model). To determine whether we could nevertheless interpret the model output and conduct the full-null model comparison, we explored the convergence problem. Since the standard deviations were very small for running trial and eye contact condition, we removed these factors from the random effect structure. The estimates of the now converging model did not differ meaningfully from the estimates of our target models. We therefore decided that we could ignore the warning messages and continue with the following steps.

**Full-null model comparison.** After successfully fitting the models, we ran likelihood ratio tests comparing the full models with the respective null models using *anova (full, null)* for each of the three dependent measures within each experiment.

**Significances and estimates of the individual fixed effects.** If the full-null model comparison indicated statistically significant effects of the predictors on the outcome, we conducted further likelihood ratio tests comparing the full model with reduced models in which the respective predictors were excluded one by one. The significance of the individual fixed effects was based on likelihood ratio tests comparing the full models with respective reduced models excluding the individual fixed effects using the *drop1*-function in R with an alpha-level of .05. The estimates and standard deviations were retrieved using the *summary* function in the *lme4* package.

**Follow-up pair-wise comparisons.** To test our hypotheses regarding the identity and location bias we furthermore tested all three possible contrasts within each of the two eye contact conditions using the R-package *emmeans* version 1.8.1-1 (Lenth et al., 2022).

# S8 – Supplementary Analyses Experiment 2

## Preregistered exploratory analyses

Like in Experiment 1, we repeated our total looking time analysis focusing on infants’ total looking time duration at the *object* instead of the entire screen. We planned this analysis as an exploratory analysis in the preregistration.

**Total looking duration at the object.** The comparison between the full model and the reduced model revealed a significant result indicating that at least one of the interactions had an impact on infants’ total looking time duration at the screen during the outcome phase (*χ^2^*(2) = 11.12, *p* = .004, see Figure S7). More specifically, the interaction between third-party eye contact and identity change outcome had a significant effect on infants’ looking time response (*χ^2^*(1) = 10.84, *p* = <.001, estimate = 0.44, *SE* = .13), with the looking times at identity change outcomes being longer in the “eye contact” condition (*M* = 4046.58 ms; *SD* = 1991.95 ms) compared to the “no eye contact” condition (*M* = 3056.63 ms, *SD* = 1695.95 ms). The interaction between third-party eye contact and location change outcome did not have a significant effect (*χ^2^*(1) = 1.72, *p* = .19, estimate = 0.18, *SE* = .14). We found a significant effect of running trial (*χ^2^*(1) = 7.45, *p* = .006, estimate = −0.22, *SE* = .07) in that infants’ looking time at the screen decreased over trials. Trial within condition (*χ^2^*(1) = 0.66, *p* = .42, estimate = 0.06, *SE* = .08) and object position (*χ^2^*(1) = 0.07, *p* = .80, estimate = −0.02, *SE* = .07) did not reveal a significant effect. Table S11 depicts the means and standard deviations of the total looking duration at the object in comparison to the means and standard deviations of both dependent measures reported in the main manuscript. Results from the post-hoc pair-wise comparisons are summarized in Table S12.

The results reflect the same memory biases as summarized in Table 3 in the main manuscript for the duration of infants’ total looking time at the screen (Experiment 2). This suggests that differences in AOI size could not explain the differences in result patterns based on the first look duration measure and the total looking time measure.

## Exploratory analyses not planned in the preregistration

We conducted the same analyses and models as in Experiment 1 to explore the data of Experiment 2 further.

**Looking times in the action phase.** First, we compared whether infants’ overall attention to the videos in the action phase different between communicative and non-communicative condition. The AOIs used for the analyses of the data in the action phase are illustrated in Figure S2.

We did not find condition differences in infants’ looking times at the overall screen (*χ^2^*(1) = 0.91, *p* = .33, estimate = −.04, *SE* = .04) and infants’ looking times at the object (*χ^2^*(1) = 0.02, *p* = .88, estimate = −35.08, *SE* = 242.52). However, infants looked longer at the actors’ faces (*χ^2^*(1) = 5.26, *p* = .02, estimate = −.10, *SE* = .04) in the “eye contact” condition compared to the “no eye contact” condition. In addition, we found a main effect of running trial, in that infants’ looking times at the actors’ faces decreased over trials (*χ^2^*(1) = 5.61, *p* = .02, estimate = −.10, *SE* = .04). The means and standard deviations of the looking times in the action phase are depicted in Table S13.

**Relation between object-related attention and encoding bias.** Second, we explored whether infants’ selective encoding of identity-relevant features in the eye contact condition was driven by their overt attention to the object during the action phase. For this purpose, we conducted the same exploratory analyses as in Experiment 1. We found that infants’ identity bias was not influenced by the looking time at the object in the action phase—neither when using the identity score based on the total looking time at the screen (*χ^2^*(1) = 0.73, *p* = .39, estimate = 236.1, *SE* = 281.0), nor when using the identity score based on the total looking time at the object (*χ^2^*(1) = 0.12, *p* = .73, estimate = −75.71, *SE* = 221.15) or the first look at the object (*χ^2^*(1) = 0.01, *p* = .90, estimate = 11.44, *SE* = 99.59). In line with the results of our preregistered main analysis, we found a significant main effect of eye contact condition in all three models, in that infants’ identity bias was significantly higher in the “eye contact” condition compared to the “no eye contact” condition. Table S14 shows the means and standard deviations of the identity bias for all three measures in both eye contact conditions.

In summary, we found that infants looked longer at the actor’s faces in the third-party “eye contact” condition compared to the “no eye contact” condition. This aligns with previous research showing that infants are highly sensitive to body orientation of third parties (face-to-face vs. back-to-back) and use it as a cue to infer social engagement from an observer perspective (Augusti, Melinder, & Gredebäck, 2010; Beier & Spelke, 2012; Goupil, Papeo, & Hochmann, 2022; Handl, Mahlberg, Norling, & Gredebäck, 2013; Thiele, Hepach, Michel, & Haun, 2021a). We did not find any indication that infants’ overt attention distribution in the action phase of Experiment 2 had caused their selective identity bias in the third-party joint attention condition. Neither was the actors’ mutual gazing to the object following eye contact related to infants’ own attention to the object during the action phase, nor was infants’ own attention to the object related to their identity bias in the outcome phase. In summary, this speaks against the assumption that infants’ object encoding was influenced by the “weirdness” of the back-to-back manipulation in the “no eye contact” condition.

# S9 – Piloting Phase

Before publishing the preregistration and starting the official data collection, we tested *N* = 7 infants (*n* = 5 female) between 9 months, 29 days, and 10 months, 8 days in a piloting phase (*M* = 307.0 days, *SD* = 3.56 days). The participants were recruited from the same database as the participants included in the final sample. The purpose of this phase was (a) to ensure that the video stimuli, the timing of the procedure, and the overall duration of the experiment were adequate for infants in the target age range, and (b) train the experimenter to conduct the calibration procedure and to apply the two-second look-away criterion. Piloting took place in October and November 2021 under the same conditions as the final study took place. Piloting was finished as soon as the experimenter felt comfortable with the technical part of the testing procedure. The piloting revealed that no adjustments were necessary regarding the timing of the procedure and the duration of the experiment.

References

Augusti, E.-M., Melinder, A., & Gredebäck, G. (2010). Look who’s talking: Pre-verbal infants’ perception of face-to-face and back-to-back social interactions. *Frontiers in Psychology, 1*. https://doi.org/10.3389/fpsyg.2010.00161

Bakdash, J. Z., & Marusich, L. R. (2017). Repeated Measures Correlation. *Frontiers in Psychology, 8*, 456. https://doi.org/10.3389/fpsyg.2017.00456

Bates, D., Maechler, M., Bolker, B., & Walker, S. (2020). *Lme4: Linear mixed-effects models using “Eigen” and S4 classes.* R-package version 1.1.15.

Beier, J. S., & Spelke, E. S. (2012). Infants’ Developing Understanding of Social Gaze: Infants’ Developing Understanding of Social Gaze. *Child Development*, *83*, 486-496. https://doi.org/10.1111/j.1467-8624.2011.01702.x

Csibra, G., Hernik, M., Mascaro, O., Tatone, D., & Lengyel, M. (2016). Statistical treatment of looking-time data. *Developmental Psychology, 52,* 521–536. https://doi.org/10.1037/dev0000083

Goupil, N., Papeo, L., & Hochmann, J. (2022). Visual perception grounding of social cognition in preverbal infants. *Infancy, 27*, 210–231. https://doi.org/10.1111/infa.12453

Handl, A., Mahlberg, T., Norling, S., & Gredebäck, G. (2013). Facing still faces: What visual cues affect infants’ observations of others? *Infant Behavior and Development, 36,* 583–586. https://doi.org/10.1016/j.infbeh.2013.06.001

Lenth, R. et al. (2022). *emmeans: Estimated Marginal Means, aka Least-Squares Means.* R- package version v. 1.8.1-1

Lo, S., & Andrews, S. (2015). To transform or not to transform: Using generalized linear mixed models to analyse reaction time data. *Frontiers in Psychology, 6.* https://doi.org/10.3389/fpsyg.2015.01171

Okumura, Y., Kobayashi, T., & Itakura, S. (2016). Eye Contact Affects Object Representation in 9-Month-Old Infants. *PLOS ONE, 11,* e0165145. https://doi.org/10.1371/journal.pone.0165145

Silverstein, P., Gliga, T., Westermann, G., & Parise, E. (2019). Probing communication-induced memory biases in preverbal infants: Two replication attempts of Yoon, Johnson and Csibra (2008). *Infant Behavior and Development, 55,* 77–87. https://doi.org/10.1016/j.infbeh.2019.03.005

Šimkovic, M., & Träuble, B. (2021). Additive and multiplicative probabilistic models of infant looking times. *PeerJ, 9,* e11771. https://doi.org/10.7717/peerj.11771

Thiele, M., Hepach, R., Michel, C., Gredebäck, G., & Haun, D. B. M. (2021). Social interaction targets enhance 13‐month‐old infants’ associative learning. *Infancy, 26,* 409–422. https://doi.org/10.1111/infa.12393

Thiele, M., Hepach, R., Michel, C., & Haun, D. (2021). Infants’ preference for social interactions increases from 7 to 13 months of age. *Child Development, 92*, 2577-2594.

Thiele, M., Hepach, R., Michel, C., & Haun, D. B. M. (2021b). Observing others’ joint attention increases 9-month-old infants’ object encoding. *Developmental Psychology, 57*, 837–850. https://doi.org/10.1037/dev0001189

Yoon, J. M. D., Johnson, M. H., & Csibra, G. (2008). Communication-induced memory biases in preverbal infants. *Proceedings of the National Academy of Sciences, 105,* 13690–13695. https://doi.org/10.1073/pnas.0804388105


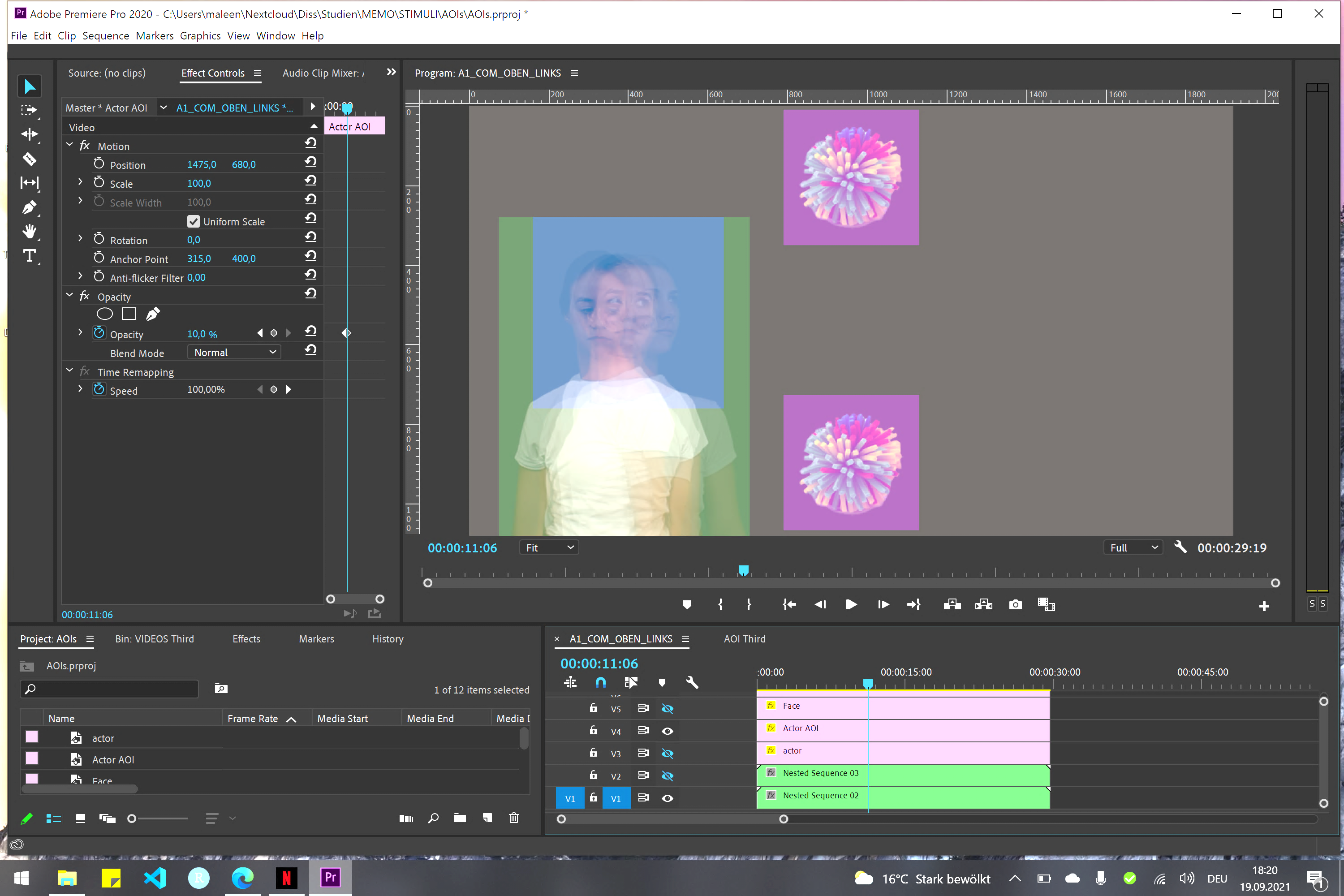


a

b


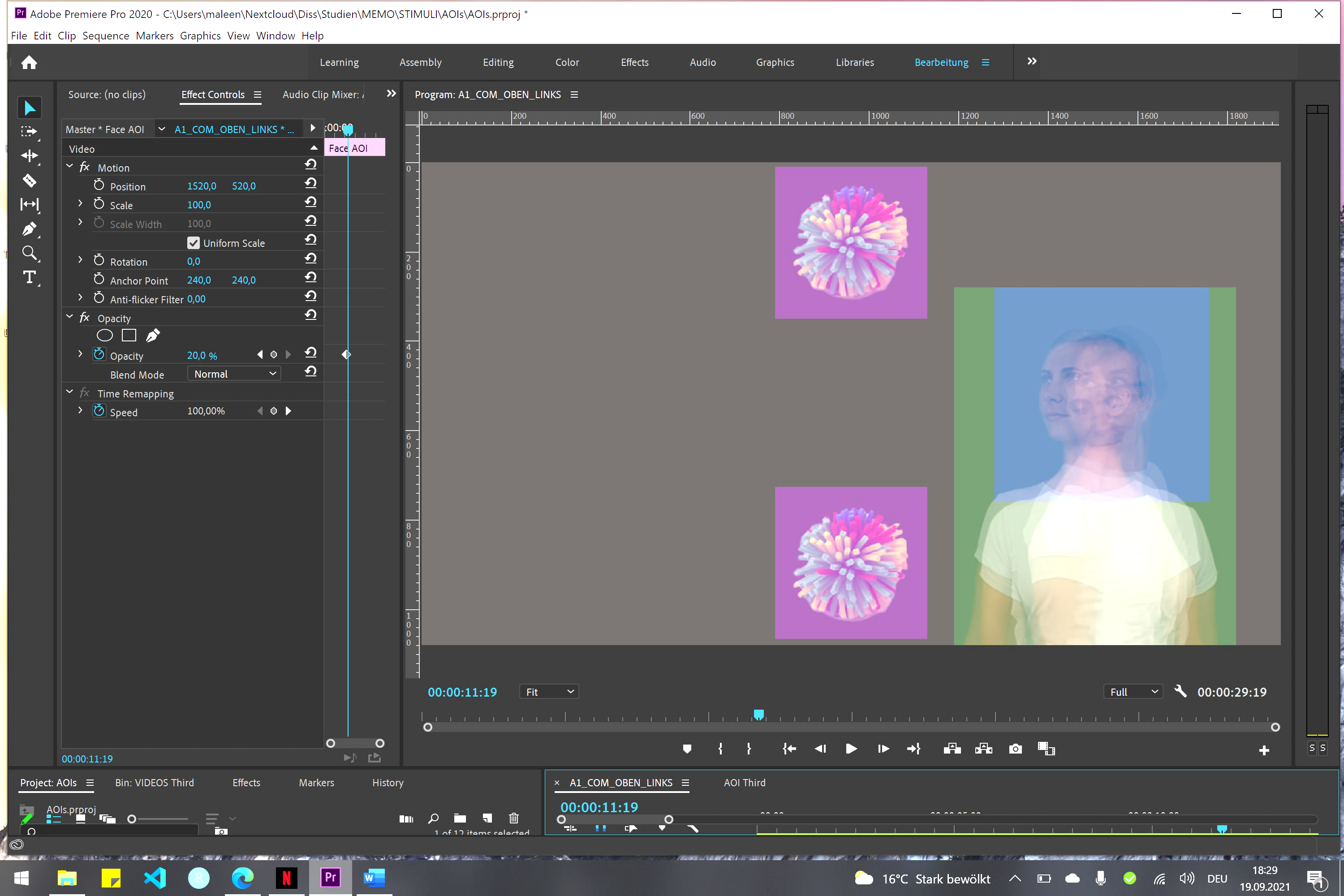


*Figure S1.* Areas of interest (AOIs) during the encoding phase of Experiment 1. (a) During trials showing the actor the right side of the object and (b) during trials showing the actor on the left side. All videos were presented in full-screen view (1920×1080 pixels). Pink area = Object AOI in the videos of the encoding and outcome phase (340×340 pixels), defined 1° visual angle larger than the maximum dimensions of the object. Blue areas = Face AOIs covering all possible head movements (480×480 pixels). Green areas = body AOIs covering all possible body movements (630×840 pixels) in the videos of the encoding phase, defined 1° visual angle larger than the areas covering all possible head and body movements from all actors in Experiment 1 and 2.


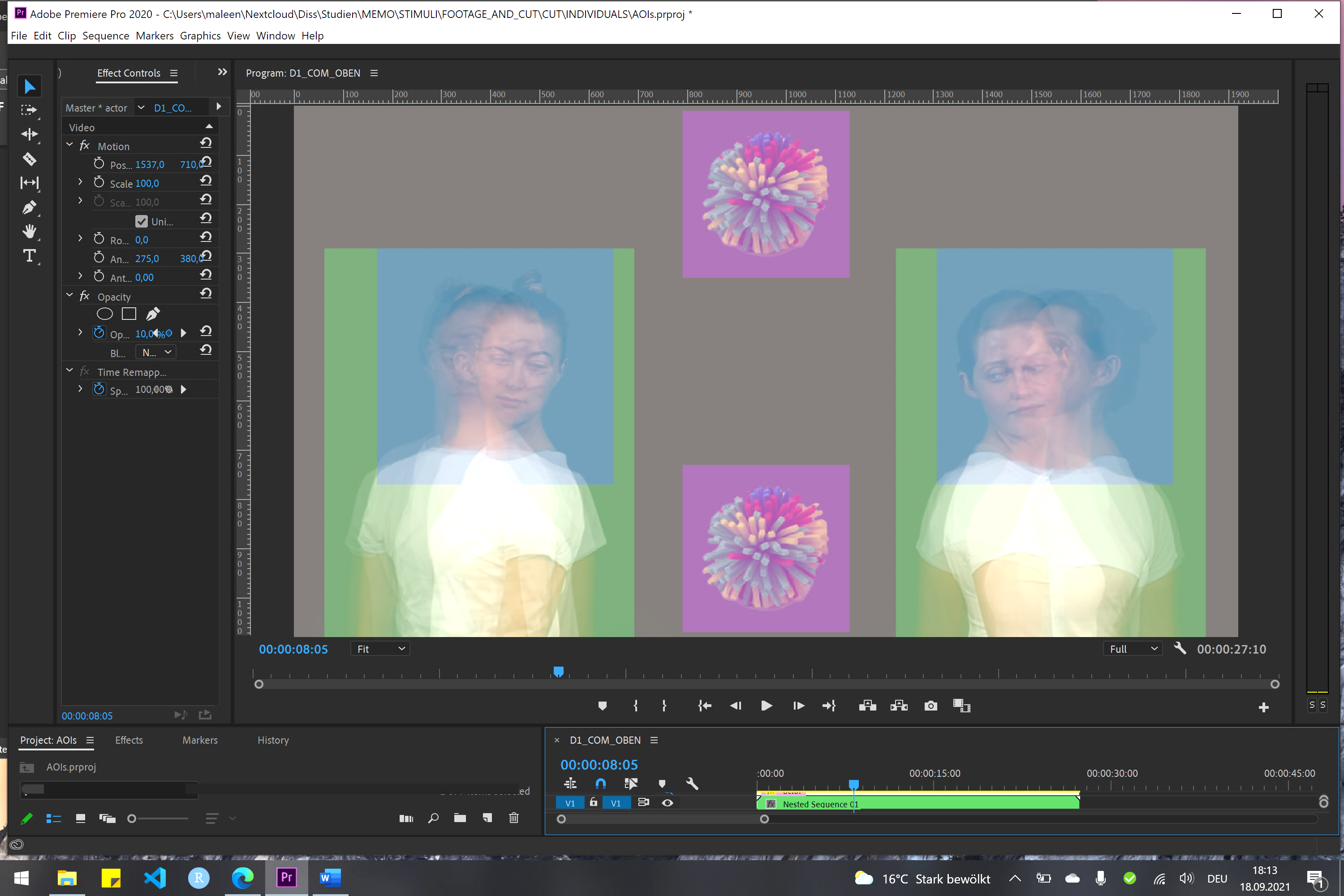


*Figure S2.* Areas of interest (AOIs) in the video stimuli of Experiment 2. All videos were presented in full-screen view (1920×1080 pixels). Pink area = Object AOI (340×340 pixels), defined 1° visual angle larger than the maximum dimensions of the object. Blue areas = Face AOIs covering all possible head movements (480×480 pixels). Green areas = Body AOIs covering all possible body movements (630×840 pixels) defined 1° visual angle larger than the areas covering all possible head and body movements from all actors in Experiment 1 and 2.


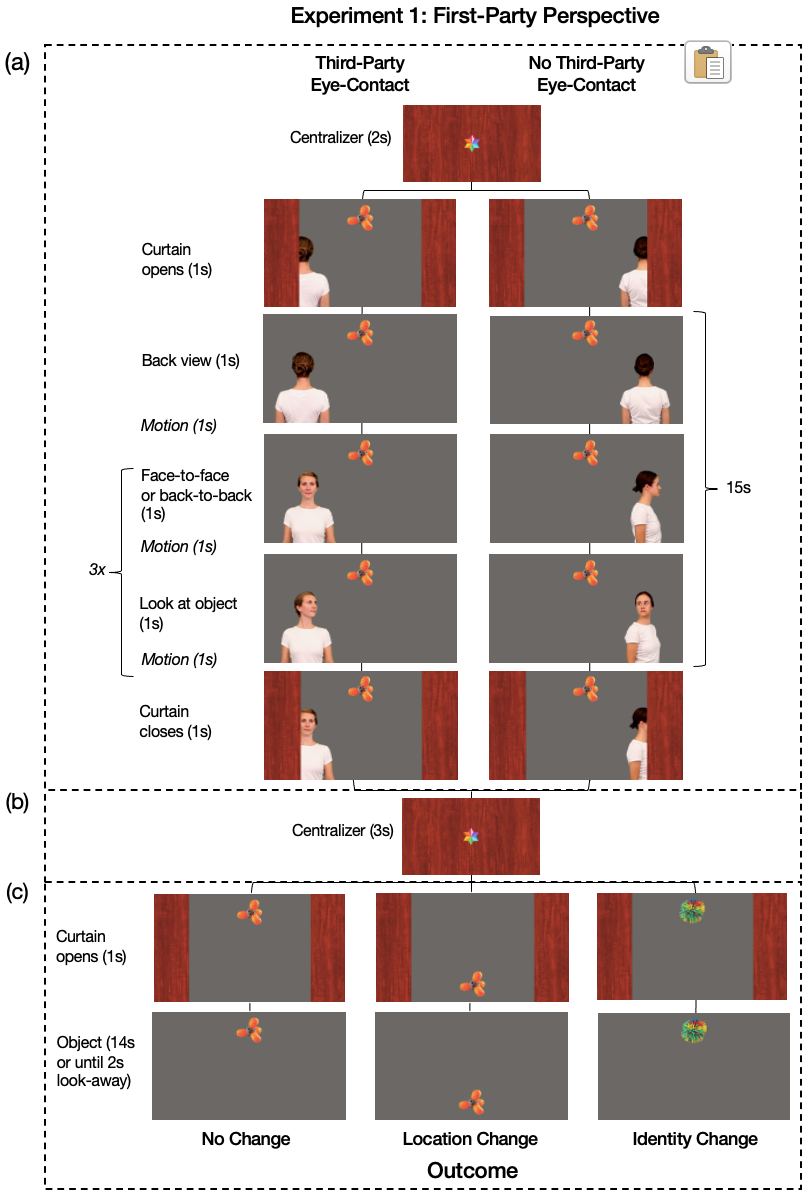


*Figure S3.* Exemplary sequence and timing of one test trial of the violation of expectation task in Experiment 1 illustrating a counterbalancing version in which the object was positioned at the upper screen position during the action phase. Every trial consisted of (a) an action phase (15s), (b) a delay phase (3s), and an outcome phase (15s or until the infant looked away for 2 consecutive seconds). Before the action and the outcome phase, an attention-getting animation (blinking star) was presented in the center of the screen. The position of the actor (left or right) was counterbalanced.


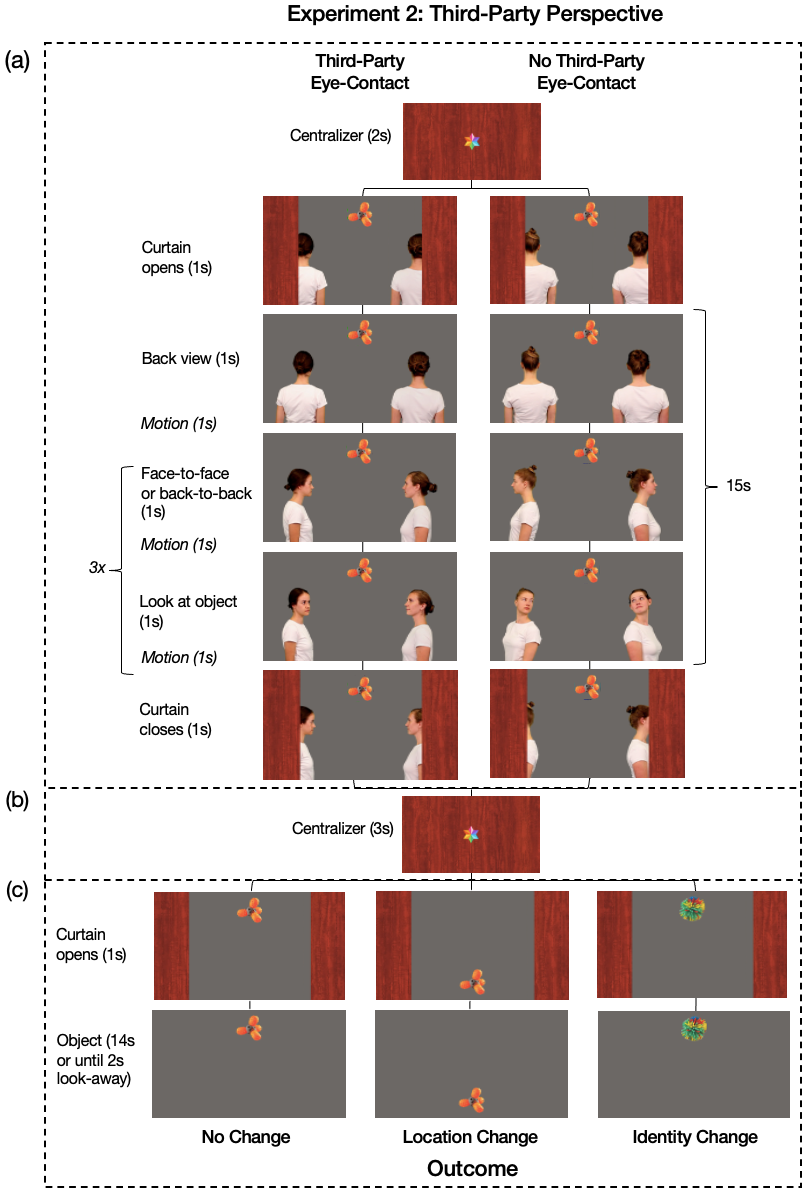


*Figure S4.* Exemplary sequence and timing of one test trial of the violation of expectation task in Experiment 2 illustrating a counterbalancing version in which the object was positioned at the upper screen position during the action phase. Every trial consisted of (a) an action phase (15s), (b) a delay phase (3s), and an outcome phase (15s or until the infant looked away for 2 consecutive seconds). Before the action and the outcome phase, an attention-getting animation (blinking star) was presented in the center of the screen.


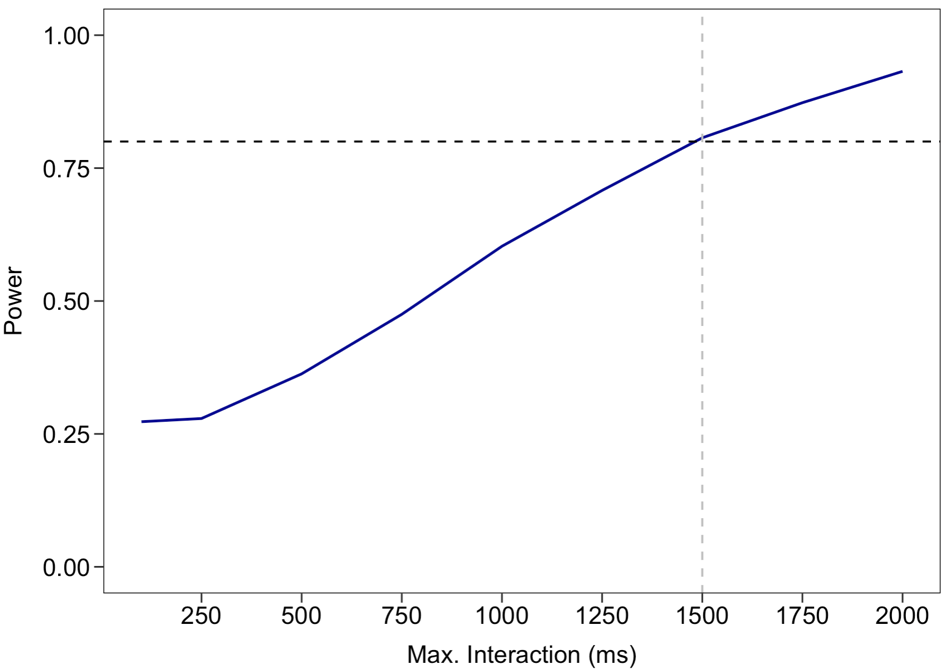


*Figure S5.* Illustration of the results of the power analysis based on 1000 simulated datasets. The y-axis depicts the maximum difference between the critical two interactions (Interaction 1: eye contact × location change; Interaction 2: eye contact × identity change).


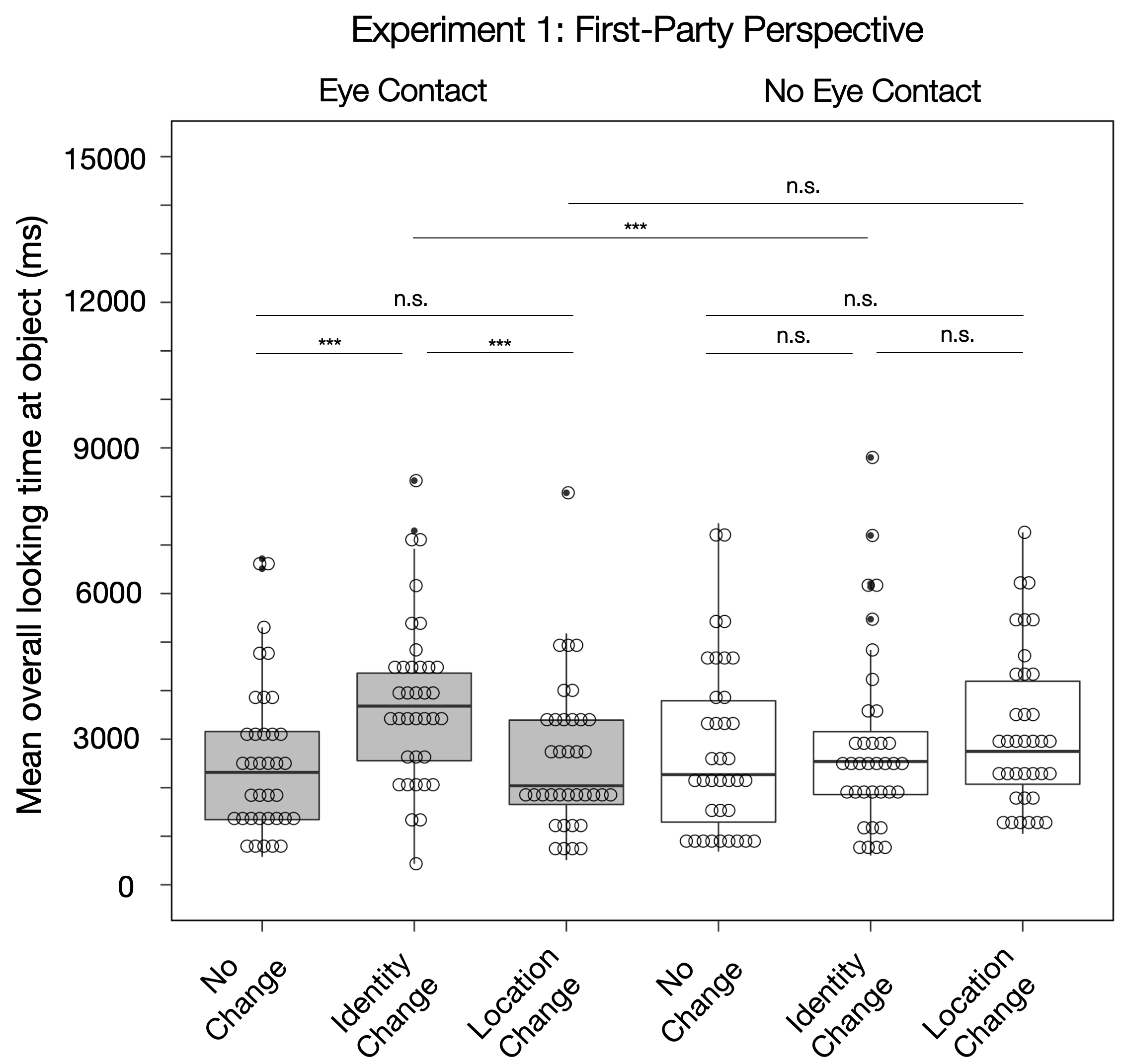


*Figure S6.* Results from Experiment 1 based on the total looking duration at the object before two consecutive seconds looking away from the object. The significances for the pair-wise comparisons were retrieved based on the R-package *emmeans* (for statistical details see Table S7). The significances across eye contact conditions represent the effect of the interactions between “eye contact × location change“ and “eye contact × identity change”.


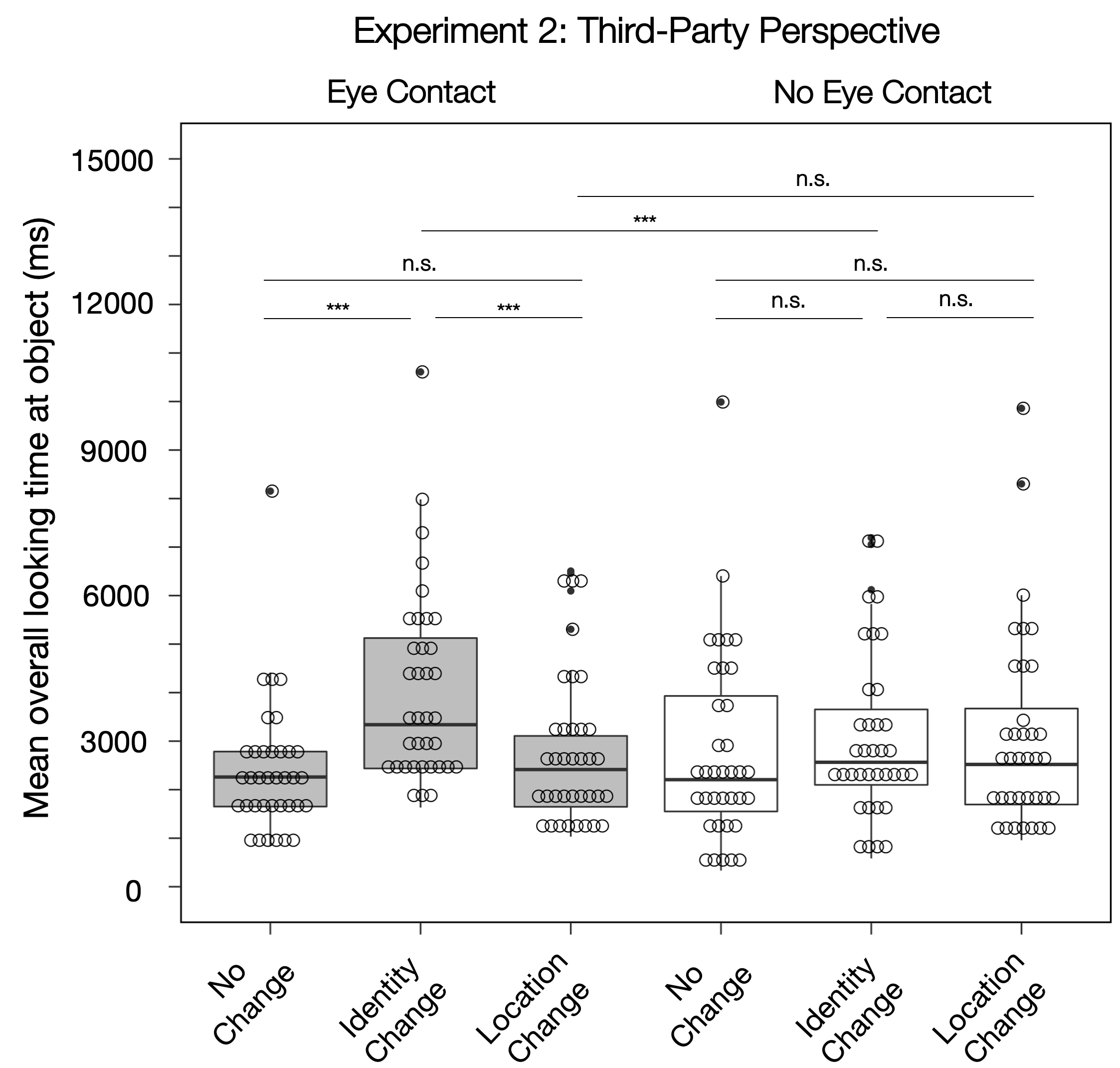


*Figure S7.* Results from Experiment 2 based on the total looking duration at the object before two consecutive seconds looking away from the object. The significances for the pair-wise comparisons were retrieved based on the R-package *emmeans* (for statistical details see Table S11). The significances across eye contact conditions represent the effect of the interactions between “eye contact × location change” and “eye contact × identity change”.


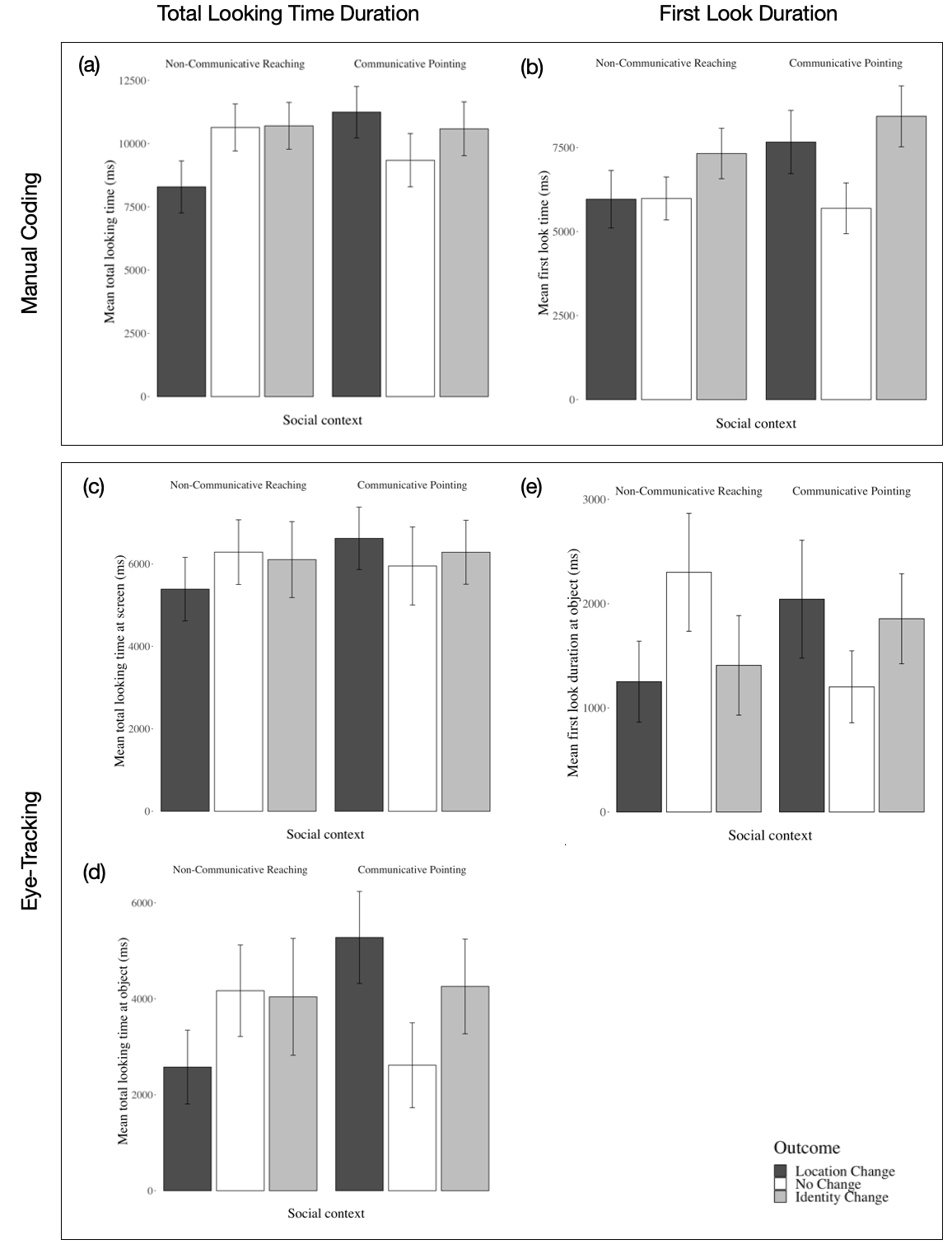


*Figure S8.* Mean duration of total looking times and first look durations based on the Experiment 1 data of the study by Silverstein et al. (2019). Comparison between the manually coded measures reported in the paper by Silverstein et al. (2019) and the corresponding eye-tracking-based measures extracted with the pre-processing script developed for our study. (a) and (b) correspond to Figures 2 and 3 in the paper by Silverstein et al. (2019) after excluding participants with bad eye-tracking data.


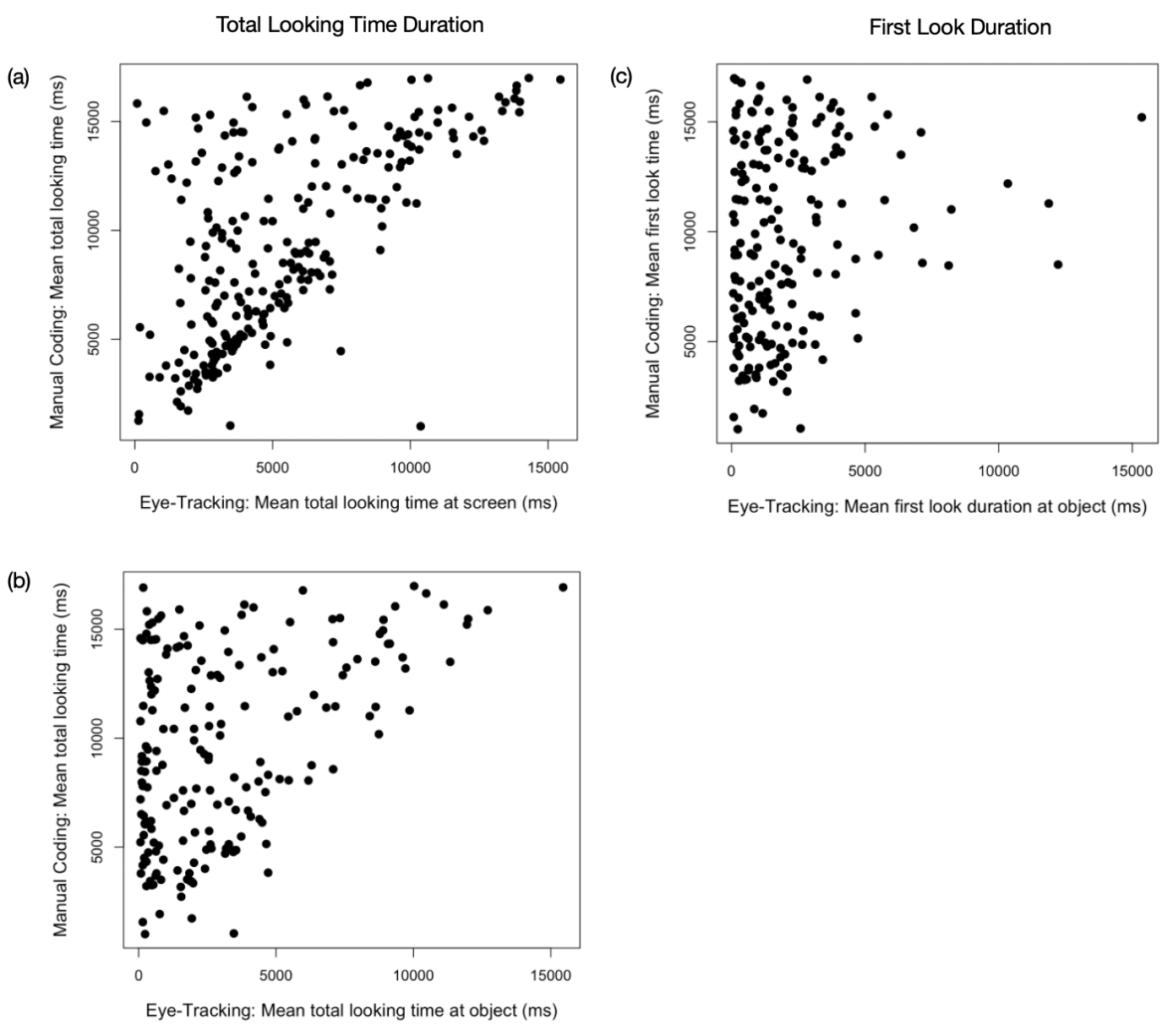


*Figure S9.* Scatterplots with individual data points representing a merged sample of all participants providing valid eye-tracking data in Experiment 1 and 2 (total *N* = 41) in the study by Silverstein et al. (2019). The two eye-tracking measures (a) mean total looking time at the screen and (b) mean looking time at the object are plotted against the manually coded mean total looking times. The eye-tracking-based (c) first look duration at the object is plotted against the corresponding first look duration measure extracted via manual coding.

Table S1

*Valid trial statistics for the six conditions in Experiment 1*

|  | Number of valid trials | | | | | |
| --- | --- | --- | --- | --- | --- | --- |
| Condition | Total | Min | Max | M*(SD)* | Infant  looking at object (action) | Infant  not looking at object  (action) |
| First-party eye contact  No change outcome | 67 | 1 | 2 | 1.86*(.35)* | 44 | 23 |
| First-party eye contact  Identity change outcome | 69 | 1 | 2 | 1.92*(.28)* | 50 | 19 |
| First-party eye contact  Location change outcome | 69 | 1 | 2 | 1.92*(.28)* | 29 | 40 |
| No first-party eye contact  No change outcome | 69 | 1 | 2 | 1.92*(.28)* | 54 | 15 |
| No first-party eye contact  Identity change outcome | 69 | 1 | 2 | 1.92*(.28)* | 46 | 23 |
| No first-party eye contact  Location change outcome | 68 | 1 | 2 | 1.89*(.32)* | 17 | 51 |
| Total | 411 | 1 | 2 | 1.90*(.30)* | 240 | 171 |

*Note.* Each individual infant could provide between 1 (min.) and 2 (max.) trials per condition. The maximum number of total trials over all infants was 72 per condition. The “Infant looking at object (action)” column represents the number of valid trials during which infants had looked at the object at all over the total duration of the action phase (i.e., fixation duration >0 ms within the object AOI). The “Infant not looking at object (action)” column represents the number of valid trials during which infants had not looked at the object at all over the total duration of the action phase (i.e., fixation duration = 0 within the object AOI).

Table S2

*Results from post-hoc pair-wise comparisons of the total looking time duration at the screen within each eye contact condition between the outcome conditions of Experiment 1*

| Compared conditions | estimate | *SE* | *z* | *p* |
| --- | --- | --- | --- | --- |
| First Party Eye Contact | | | | |
| No Change – Identity Change | –.28 | .08 | –3.39 | .002** |
| No Chance – Location Change | –.03 | .09 | –.32 | 1.0 |
| Identity Change – Location Change | .26 | .08 | 3.08 | .006** |
| No First-Party Eye Contact | | | | |
| No Change – Identity Change | –.03 | .08 | –.32 | 1.0 |
| No Chance – Location Change | –.01 | .09 | –.15 | 1.0 |
| Identity Change – Location Change | .01 | .08 | .16 | 1.0 |

*Note.* Results are based on the pairwise contrasts between the estimated marginal means of all conditions, inferred from the main GLMM fitted to the data by using the R-package *emmeans*. P-values are adjusted via Bonferroni correction within each eye contact condition for three tests.

Table S3

*Results from post-hoc pair-wise comparisons of the first look duration at the object within each eye contact condition between the outcome conditions of Experiment 1*

| Compared conditions | estimate | *SE* | *z* | *p* |
| --- | --- | --- | --- | --- |
| First Party Eye Contact | | | | |
| No Change – Identity Change | –.67 | .07 | –9.39 | <.001*** |
| No Chance – Location Change | –.27 | .07 | –3.70 | <.001*** |
| Identity Change – Location Change | .40 | .07 | 5.69 | <.001*** |
| No First-Party Eye Contact | | | | |
| No Change – Identity Change | –.27 | .07 | –3.81 | <.001*** |
| No Chance – Location Change | –.29 | .07 | –4.0 | <.001*** |
| Identity Change – Location Change | –.02 | .07 | –0.27 | 1.0 |

*Note.* Results are based on the pairwise contrasts between the estimated marginal means of all conditions, inferred from the main GLMM fitted to the data by using the R-package *emmeans*. P-values are adjusted via Bonferroni correction within each eye contact condition.

Table S4

*Valid trial statistics for the six conditions in Experiment 2*

|  | Number of valid trials | | | | | |
| --- | --- | --- | --- | --- | --- | --- |
| Condition | Total | Min | Max | M*(SD)* | Infant  looking at object (action) | Infant  not looking at object  (action) |
| Third-party eye contact  No change outcome | 65 | 1 | 2 | 1.81*(.40)* | 43 | 22 |
| Third-party eye contact  Identity change outcome | 66 | 1 | 2 | 1.83*(.38)* | 47 | 19 |
| Third-party eye contact  Location change outcome | 70 | 1 | 2 | 1.94*(.23)* | 25 | 45 |
| No third-party eye contact  No change outcome | 66 | 1 | 2 | 1.83*(.38)* | 42 | 24 |
| No third-party eye contact  Identity change outcome | 69 | 1 | 2 | 1.92*(.28)* | 34 | 35 |
| No third-party eye contact  Location change outcome | 68 | 1 | 2 | 1.89*(.32)* | 21 | 47 |
| Total | 404 | 1 | 2 | 1.87*(.34)* | 212 | 192 |

*Note.* Each individual infant could provide between 1 (min.) and 2 (max.) trials per condition. The maximum number of total trials over all infants was 72 per condition. The “Infant looking at object (action)” column represents the number of valid trials during which infants had looked at the object at all over the total duration of the action phase (i.e., fixation duration >0 ms within the object AOI). The “Infant not looking at object (action)” column represents the number of valid trials during which infants had not looked at the object at all over the total duration of the action phase (i.e., fixation duration = 0 within the object AOI).

Table S5

*Results from post-hoc pair-wise comparisons of the total looking time duration at the screen within each eye contact condition between the outcome conditions of Experiment 2*

| Compared conditions | estimate | *SE* | *z* | *p* |
| --- | --- | --- | --- | --- |
| Third-Party Eye Contact | | | | |
| No Change – Identity Change | –.33 | .09 | –3.82 | <.001*** |
| No Chance – Location Change | –.06 | .08 | –0.75 | 1.0 |
| Identity Change – Location Change | .26 | .08 | 3.20 | .004** |
| No Third-Party Eye Contact | | | | |
| No Change – Identity Change | –.02 | .08 | –0.27 | 1.0 |
| No Chance – Location Change | .08 | .09 | 0.90 | 1.0 |
| Identity Change – Location Change | .10 | .08 | 1.18 | .71 |

*Note.* Results are based on the pairwise contrasts between the estimated marginal means of all conditions, inferred from the main GLMM fitted to the data by using the R-package *emmeans*. P-values are adjusted via Bonferroni correction within each eye contact condition.

Table S6

*Results from post-hoc pair-wise comparisons of the first look duration at the object within each eye contact condition between the outcome conditions of Experiment 2*

| Compared conditions | estimate | *SE* | *z* | *p* |
| --- | --- | --- | --- | --- |
| Third-Party Eye Contact | | | | |
| No Change – Identity Change | –.77 | .08 | –10.08 | <.001*** |
| No Chance – Location Change | –.32 | .07 | –4.29 | <.001*** |
| Identity Change – Location Change | .45 | .08 | 5.99 | <.001*** |
| No Third-Party Eye Contact | | | | |
| No Change – Identity Change | –.09 | .08 | –1.22 | .66 |
| No Chance – Location Change | –.24 | .08 | –3.14 | .005** |
| Identity Change – Location Change | –.15 | .07 | –1.99 | .14 |

*Note.* Results are based on the pairwise contrasts between the estimated marginal means of all conditions, inferred from the main GLMM fitted to the data by using the R-package *emmeans*. P-values are adjusted via Bonferroni correction within each eye contact condition.

Table S7

*Means and standard deviations (ms) of the total looking time at the object in comparison to the two dependent measures reported in the main manuscript for the six conditions in Experiment 1*

| Condition | Duration of total looking time at the screen | Duration of total looking time at the object | Duration of first look at  the object |
| --- | --- | --- | --- |
| First-party eye contact  No change outcome | 3829.22 *(1788.88)* | 2548.65  *(1579.43)* | 1020.79  *(476.74)* |
| First-party eye contact  Identity change outcome | 4745.54 *(1908.005)* | 3765.78  *(1694.58)* | 2078.18  *(978.20)* |
| First-party eye contact  Location change outcome | 3598.14 *(2000.38)* | 2586.32  *(1526.92)* | 1336.17  *(625.62)* |
| No first-party eye contact  No change outcome | 3826.60 *(2303.40)* | 2779.38  *(1788.95)* | 1028.00  *(472.07)* |
| No first-party eye contact  Identity change outcome | 3913.35 *(2350.92)* | 2904.26  *(1875.43)* | 1416.11  *(734.48)* |
| No first-party eye contact  Location change outcome | 4048.44 *(1794.07)* | 3165.67  *(1611.46)* | 1472.57  *(734.03)* |

*Note.* The columns “Duration of total looking time at the screen” and “Duration of first look at the object” correspond to Table 1 in the main manuscript.

Table S8

*Results from post-hoc pair-wise comparisons of the total looking time duration at the object within each eye contact condition between the outcome conditions of Experiment 1*

| Compared conditions | estimate | *SE* | *z* | *p* |
| --- | --- | --- | --- | --- |
| First Party Eye Contact | | | | |
| No Change – Identity Change | –.50 | .09 | –5.31 | <.001*** |
| No Chance – Location Change | –.14 | .09 | –1.51 | .39 |
| Identity Change – Location Change | .35 | .09 | 3.85 | <.001*** |
| No First-Party Eye Contact | | | | |
| No Change – Identity Change | –.04 | .09 | –.46 | 1.0 |
| No Chance – Location Change | –.04 | .09 | –.41 | 1.0 |
| Identity Change – Location Change | .003 | .09 | .03 | 1.0 |

*Note.* Results are based on the pairwise contrasts between the estimated marginal means of all conditions, inferred from the main GLMM fitted to the data by using the R-package *emmeans*. P-values are adjusted via Bonferroni correction within each eye contact condition.

Table S9

*Means and standard deviations (ms) for looking times during the action phase in Experiment 1*

|  | Action Phase Condition | |  |
| --- | --- | --- | --- |
|  | First-Party Eye Contact | No First-Party Eye Contact | |
| LT Screen AOI (n.s.) | 11008.28 *(2370.19)* | 11064.36 *(2093.45)* | |
| LT Object AOI (n.s.) | 917.57 *(1138.47)* | 836.94 *(1004.16)* | |
| LT Face AOIs (n.s.) | 9298.49 *(2592.63)* | 9486.72 *(2468.56)* | |

*Note.* The looking times represent the sum of fixation durations within the corresponding area of interest (AOI) in milliseconds (ms). The total video duration was 15000 ms. The significances depicted in parentheses illustrate significances of the fixed effect of eye contact, retrieved from a model described in more detail in section S5.

Table S10

*Means and standard deviations (ms) for the identity bias for all three outcome measures in Experiment 1*

|  | Action Phase Condition | |  |
| --- | --- | --- | --- |
|  | First-Party Eye  Contact | No First-Party Eye Contact | |
| Total looking time at screen | 916.32 *(2275.60)* | 86.75 *(2811.20)* | |
| Total looking time at object | 1217.13*(2066.49)* | 124.89 *(2279.25)* | |
| First look duration at object | 1057.39 *(743.68)* | 388.11 *(597.60)* | |

*Note.* An identity bias was defined as the difference between looking time response in the no change condition and the identity change condition in the outcome phase. The higher the mean looking time difference, the stronger the bias toward the respective outcome change.

Table S11

*Means and standard deviations (ms) of the total looking time at the object in comparison to the two dependent measures reported in the main manuscript for the six conditions in Experiment 2*

| Condition | Duration of total looking time at  the screen | Duration of total looking time at the object | Duration of first look at  the object |
| --- | --- | --- | --- |
| Third-party eye contact  No change outcome | 3657.03  *(1890.30)* | 2403.78  *(1364.34)* | 1102.71  *(450.25)* |
| Third-party eye contact  Identity change outcome | 5036.75  *(2225.87)* | 4046.58  *(1991.95)* | 2490.96  *(1169.52)* |
| Third-party eye contact  Location change outcome | 3766.56  *(2189.52)* | 2732.75  *(1508.87)* | 1518.24  *(557.08)* |
| No third-party eye contact  No change outcome | 3795.79  *(2248.37)* | 2791.99  *(1981.37)* | 1282.42  *(639.89)* |
| No third-party eye contact  Identity change outcome | 3985.07  *(2137.05)* | 3056.63  *(1695.95)* | 1482.78  *(702.26)* |
| No third-party eye contact  Location change outcome | 3857.24  *(2162.68)* | 3079.03  *(2018.99)* | 1696.54  *(713.34)* |

*Note.* The columns “Duration of total looking time at the screen” and “Duration of first look at the object” correspond to Table 2 in the main manuscript.

Table S12

*Results from post-hoc pair-wise comparisons of the total looking time duration at the object within each eye contact condition between the outcome conditions of Experiment 2*

| Compared conditions | estimate | *SE* | *z* | *p* |
| --- | --- | --- | --- | --- |
| Third-Party Eye Contact | | | | |
| No Change – Identity Change | –.53 | .09 | –5.63 | <.001*** |
| No Chance – Location Change | –.19 | .09 | –2.10 | .10 |
| Identity Change – Location Change | .33 | .09 | 3.65 | <.001*** |
| No Third-Party Eye Contact | | | | |
| No Change – Identity Change | –.08 | .09 | –.89 | 1.0 |
| No Chance – Location Change | –.02 | .10 | –.18 | 1.0 |
| Identity Change – Location Change | .07 | .09 | .72 | 1.0 |

*Note.* Results are based on the pairwise contrasts between the estimated marginal means of all conditions, inferred from the main GLMM fitted to the data by using the R-package *emmeans*. P-values are adjusted via Bonferroni correction within each eye contact condition.

Table S13

*Means and standard deviations (ms) for looking times during the action phase in Experiment 2*

|  | Action Phase Condition | |  |
| --- | --- | --- | --- |
|  | Third-Party Eye Contact | No Third-Party Eye Contact | |
| LT Screen AOI (n.s.) | 10775.78 *(2333.41)* | 11094.19 *(2431.87)* | |
| LT Object AOI (n.s.) | 943.34 *(1211.71)* | 812.44 *(1147.47)* | |
| LT Face AOIs (*) | 8773.71 *(2774.82)* | 9411.03 *(2709.24)* | |

*Note.* The looking times represent the sum of fixation durations within the corresponding area of interest (AOI) in milliseconds (ms). The total video duration was 15000 ms. The significances depicted in parentheses illustrate significances of the fixed effect of eye contact, retrieved from a model described in more detail in section S5.

Table S14

*Means and standard deviations (ms) for the identity bias for all three outcome measures in Experiment 2*

|  | Action Phase Condition | |  |
| --- | --- | --- | --- |
|  | Third-Party Eye Contact | No Third-Party Eye Contact | |
| Total looking time at screen | 1379.72 *(2695.06)* | 189.28 *(1975.71)* | |
| Total looking time at object | 264.64 *(1792.59)* | 1642.81 *(1923.28)* | |
| First look duration at object | 1388.25 *(1137.99)* | 200.36 *(699.88)* | |

*Note.* An identity bias was defined as the difference between looking time response in the no change condition and the identity change condition in the outcome phase. The higher the mean looking time difference, the stronger the bias toward the identity outcome change.
